# UFL1 promotes antiviral immune response by maintaining STING stability independent of UFMylation

**DOI:** 10.1038/s41418-022-01041-9

**Published:** 2022-07-23

**Authors:** Yijie Tao, Shulei Yin, Yang Liu, Chunzhen Li, Yining Chen, Dan Han, Jingyi Huang, Sheng Xu, Zui Zou, Yizhi Yu

**Affiliations:** 1 National Key Laboratory of Medical Immunology and Institute of Immunology, Naval Medical University, Shanghai, 200433 China; 2 Department of Anesthesiology, Changzheng Hospital, Second Affiliated Hospital of Naval Medical University, Shanghai, 200003 China; 3 School of Anesthesiology, Naval Medical University, 168 Changhai Road, Shanghai, 200433 China

**Keywords:** Cell death and immune response, Ubiquitylation

## Abstract

The precise regulation of STING homeostasis is essential for its antiviral function. Post-translational modification, especially ubiquitination, is important for the regulation of STING homeostasis. Previous studies have focused on how STING is degraded, but little is known about its maintenance. Here, we show that UFM1 specific ligase UFL1 promotes innate immune response by maintaining STING expression independent of UFMylation. Mechanistically, UFL1 inhibits TRIM29 to interact with STING, thereby reducing its ubiquitination at K338/K347/K370 and subsequent proteasomal degradation. DNA virus infection reduces the UFL1 expression, which may promote STING degradation and facilitate viral expansion. Our study identifies UFL1 as a crucial regulator for the maintenance of STING stability and antiviral function, and provides novel insights into the mechanistic explanation for the immunological escape of DNA virus.

## Introduction

Innate immune system is the first line defense against the invasion of pathogenic microorganisms. Its activation depends on pattern recognition receptors (PPRs), such as Toll-like receptors (TLRs), RIG-I like receptors (RLRs), and nucleotide binding oligomerization domain-like receptors (NLRs), to discern pathogen-associated molecular patterns (PAMPs) or danger-associated molecular patterns (DAMPs) and activate downstream signal pathways. Among these, stimulator of interferon genes (STING) plays a key role in antiviral innate immune responses. Cyclic guanosate-adenylate synthase (cGAS)–a major sensor of DNA, can recognize pathogen genomic, mitochondrial DNA (mtDNA) [1, 2], or cyclic dinucleotides (CDNs) [3] to catalyze the synthesis of cyclic GMP-AMP (cGAMP) [4, 5]. Whereafter, cGAMP functions as a second messenger to interact with STING and promotes its dimerization [6]. Activated STING recruits TANK-binding kinase 1 (TBK1), which phosphorylates STING and the transcription factor IFN regulatory factor 3 (IRF3) subsequently. Phosphorylated IRF3 dimerizes and translocates into the nucleus and works together with nuclear factor κB (NF-κB) to turn on the expression of type I interferon (IFN) and inflammatory cytokines, leading to antiviral immune responses [7]. As the key adaptor of IRF3 and NF-κB signal pathways, STING is crucial for effective innate immune responses.

Post-translational modification (PTM) refers to the covalent or enzymatic modification that occurs during or after protein synthesis. More than 200 types of PTMs have been discovered. Among them, ubiquitination is one of the most common PTMs, except for glycosylation and phosphorylation [8]. Ubiquitination is essential for potent production of the type I IFN and proinflammatory cytokines to combat the pathogens [9]. Different types of ubiquitination have also been found in the regulation of STING homeostasis. Studies showed that K48-linked ubiquitination of STING by ring finger 5 (RNF5), tripartite motif protein 30a (TRIM30a) and tripartite motif protein 29 (TRIM29) can promote the proteasomal degradation of STING [10–13], thereby reducing the antiviral innate immune response. In addition, K63-linked ubiquitination by tripartite motif protein 32 (TRIM32) and tripartite motif protein 56 (TRIM56) can promote the formation of STING and TBK1 complex [14, 15]. Autocrine motility factor receptor (AMFR) and insulin induced gene 1 (INSIG1) complex can also be recruited to STING, promoting K27-linked ubiquitination and activation of downstream signal pathways [16].

Recently, a number of Ub-like proteins (UBLs), such as small Ub-like modifier (SUMO) [17], neural precursor cell-expressed and developmentally-downregulated 8 (NEDD8) [18], interferon-stimulated gene 15 (ISG15) [19] and ubiquitin fold modifier 1 (UFM1) [20] were also found to be covalently conjugated to their targets through a series of enzymatic reactions. Among them, UFM1 is one of the newly discovered UBLs in recent years. Similar to ubiquitin, UFM1 is conjugated to its target proteins by a three-step enzymatic reaction, which contains: the UFM1-activating enzyme E1, ubiquitin-like modifier-activating enzyme 5 (UBA5); E2, the UFM1-conjugating enzyme 1 (UFC1); and E3, the UFM1-specific ligase 1 (UFL1) [21]. UFL1 is the sole E3 ligase of UFMylation till now. UFMylation modification mediated by UFL1 is involved in the regulation of many important physiological (ER stress, development and differentiation of blood progenitors, etc.) [22, 23] and pathological processes (DNA damage, heart failure, cancer, inflammation, etc.) [24–27]. However, the function of UFL1 in antiviral innate immunity is poorly understood.

In this report, UFL1 was found to be significantly decreased in primary macrophages after DNA virus infection. Mice with *Ufl1*-deficent macrophages are impaired in defense against DNA virus (HSV-1) infection, with less type I IFN and inflammation cytokines production. Furthermore, the K48-linked ubiquitination and proteasomal degradation of STING are reduced in cells deficient of UFL1. Our research discovered a novel function of UFL1 in antiviral innate immunity independent of UFMylation and provided an explanation of the mechanisms of DNA virus immune escape.

## Results

### UFL1 promotes antiviral innate immunity

**Fig. 1:**
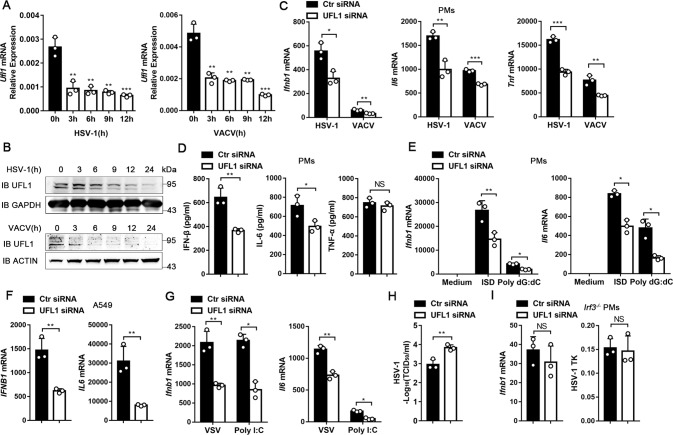
UFL1 promotes antiviral innate immunity. **A**, **B** *Ufl1* expression in PMs infected with HSV-1 or VACV detected by qRT-PCR (**A**) and Western blot (**B**). **C** *Ifnb1*, *Il6* or *Tnf* mRNA expression in PMs infected with HSV-1 or VACV. **D** IFN-β and cytokines production in supernatants of PMs 24 hr post HSV-1 infection. **E** *Ifnb1* and *Il6* mRNA expression in PMs transfected with ISD or Poly dG:dC. **F** *IFNB1* and *IL6* mRNA expression in A549 cells transfected with Poly dA:dT. **G** *Ifnb1* and *Il6* mRNA expression in PMs infected with VSV or transfected with Poly I:C. **H** HSV-1 titers in supernatants of PMs by TCID50 assay. **I** *Ifnb1* mRNA and HSV-1 TK RNA expression in *Irf3*^*−/−*^ PMs infected with HSV-1. Data are presented as means ± SD from three independent experiments. *P < 0.05, **P < 0.01, ***P < 0.001.

In order to identify the function of UFMylation in the innate immune response against DNA viral infection, the expression of UFL1, which is the only E3-like ligase of UFM1, was analyzed in peritoneal macrophages infected with herpes simplex virus (HSV-1) or vaccinia virus (VACV). UFL1 decreased dramatically and quickly after infection (Fig. 1A, B and Supplementary Fig. 1A). These results suggest that UFL1 may play an important role in antiviral innate immunity. Furthermore, the same phenomenon was also observed in *Irf3*^*−/−*^ macrophages (Supplementary Fig. 1B) and *cgas*^*−/−*^ L929 cells (Supplementary Fig. 1C), which means the cGAS-STING pathway and type I IFNs were not indispensable for the regulation of UFL1 expression. Previous reports found that TLRs play a critical role in the recognition of HSV-1 during viral entry and replication. Therefore, we hypothesized whether the down-regulation of UFL1 was related to TLRs-triggered signaling pathways. Indeed, stimulation with ligands for TLR2, TLR3 and TLR4 could downregulated UFL1 (Supplementary Fig. 1D). Then we further investigated the signal pathways responsible for the down-regulation. Different inhibitors of NF-κB and mitogen-activated protein kinase cascades (MAPK) signaling pathways were used and only the inhibitor of NF-κB could rescue UFL1 down-regulation (Supplementary Fig. 1E). The above results suggest that the down-regulation of UFL1 might related to TLRs triggered NF-κB pathway during HSV-1 infection.

In order to investigate the effect of UFL1, we knocked it down in peritoneal macrophages (Supplementary Fig. 1F) and found the mRNA expression of interferon beta 1 (*Ifnb1*), interleukin 6 (*Il6*) and tumor necrosis factor (*Tnf*) significantly decreased after HSV-1 or VACV infection (Fig. 1C). The same phenomenon was also observed in bone marrow-derived macrophages (BMDMs) (Supplementary Fig. 1G), and further confirmed by ELISA assay (Fig. 1D). UFL1 knockdown also led to obviously less *Ifnb1* and *Il6* mRNA production in response to intracellular transfection of ISD and Poly dG:dC (Fig. 1E). Furthermore, the effect of UFL1 was confirmed in human A549 cells (Fig. 1F and Supplementary Fig. 1H). UFL1 knockdown also led to obviously less *Ifnb1* and *Il6* mRNA production in response to RNA virus vesicular stomatitis virus (VSV) and intracellular transfection of Poly I:C (Fig. 1G). These data suggest that UFL1 is a positive regulator of antiviral innate immunity and promotes type I interferon production.

Correspondingly, HSV-1 titer and replication were significantly increased after UFL1 knocked down (Fig. 1H and Supplementary Fig. 1I, J). To fully illustrate whether the promotion of DNA viral loads by UFL1 knockdown was dependent on reduced type I IFN production, *Irf3*^*−/−*^ macrophages were used. In the absence of IRF3-type I IFN pathway, the effect of UFL1 knockdown on viral replication disappeared, suggesting UFL1 regulates DNA viral replication through type I IFN production (Fig. 1I and Supplementary Fig. 1K). However, different from DNA virus, the VSV RNA loads and *Ifnb1* mRNA production were both decreased in macrophages after silencing UFL1 (Fig. 1G and Supplementary Fig. 1L), which suggested UFL1 might preferentially regulate VSV replication, and the reduced VSV loads led to less IFN-β production. To verify this, we further detected the VSV replication in *Irf3*^*−/−*^macrophages and found that UFL1 deficiency decreased VSV loads as well (Supplementary Fig. 1M), suggesting IFN is not responsible for the down-regulation of viral loads. The above data suggest that UFL1 regulates IFN production indirectly through interfering viral replication in RNA viral infection.

### UFL1 deficiency suppresses antiviral innate immunity

**Fig. 2:**
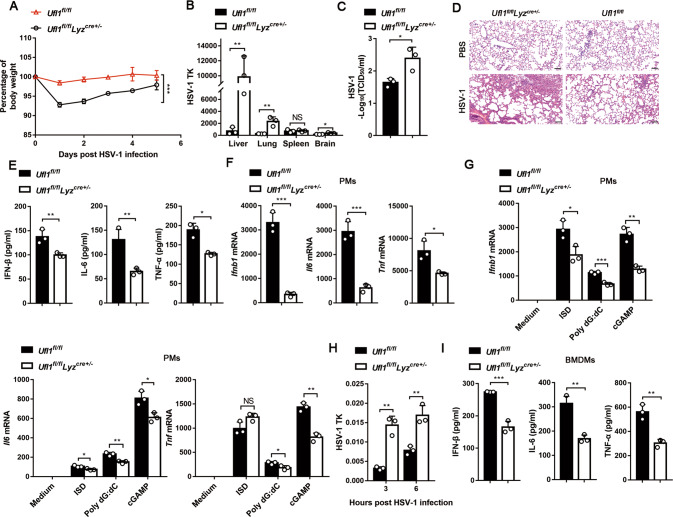
Loss of UFL1 in macrophages drives mice more susceptible to HSV-1 infection. **A** Weight loss of *Ufl1*^*fl/fl*^ and *Ufl1*^*fl/fl*^*Lyz*^*cre+/***−**^ mice after intraperitoneal injection of HSV-1 (1 × 10^8^ pfu/g). **B–E** HSV-1 TK RNA expression (**B**), HSV-1 titers in lung homogenates (**C**), H&E of lung (**D**) and cytokines production in the serum (**E**) from *Ufl1*^*fl/fl*^ and *Ufl1*^*fl/fl*^*Lyz*^*cre+/***−**^ mice in response to i.v. infection with HSV-1. The scale bar is 100 µm (**D**). **F**, **G** *Ifnb1*, *Il6*, or *Tnf* mRNA expression in PMs from *Ufl1*^*fl/fl*^ and *Ufl1*^*fl/fl*^*Lyz*^*cre+/***−**^ mice infected with HSV-1 (**F**) or transfected with cGAMP and exogenous nucleic acid stimulations (**G**). **H** HSV-1 TK RNA expression in PMs from *Ufl1*^*fl/fl*^ and *Ufl1*^*fl/fl*^*Lyz*^*cre+/***−**^ mice infected with HSV-1. **I** IFN-β and cytokines production in supernatants of BMDMs from *Ufl1*^*fl/fl*^ and *Ufl1*^*fl/fl*^*Lyz*^*cre+/***−**^ mice infected with HSV-1 for 24 h. Data are presented as means ± SD from three independent experiments. *P < 0.05, **P < 0.01, ***P < 0.001.

To investigate the significance of UFL1 in host antiviral innate immune response in vivo, mice with specific deletion of *Ufl1* in macrophages (*Ufl1*^*fl/fl*^*Lyz*^*cre+/***−**^) were developed (Supplementary Fig. 2A). *Ufl1*^*fl/fl*^*Lyz*^*cre+/***−**^ mice (hereafter referred as *Ufl1*^*−/−*^) were comparable to littermate control mice in terms of body and spleen weight and did not exhibit any obvious differences in the development of myeloid cell populations (Supplementary Fig. 2B–D). Upon infection with HSV-1, *Ufl1*^*−/−*^ mice generated more weight loss than littermates (Fig. 2A). HSV-1 replication was also significantly increased in liver, lung and brain from *Ufl1*^*−/−*^ mice (Fig. 2B). Moreover, HSV-1 titer elevated obviously in the lung homogenates from *Ufl1*^*−/−*^ mice (Fig. 2C). Accordingly, more neutrophil infiltration, alveolar hemorrhage and alveolar wall thickness were found in the lung of *Ufl1*^*−/−*^ mice (Fig. 2D). In addition, *Ufl1*^*−/−*^ mice produced lower concentrations of IFN-β, IL-6 and TNF-α in serum than littermates after HSV-1 infection (Fig. 2E). These data demonstrate that the innate antiviral response in *Ufl1*^*−/−*^ mice is effectively attenuated.

The effect of *Ufl1* deficiency in macrophages was also confirmed in vitro. After infection with HSV-1, cGAMP and exogenous nucleic acid stimulations, lower *Ifnb1* and *Il6* mRNA expression was found in the *Ufl1*^*−/−*^ peritoneal macrophages (Fig. 2F, G). The same phenomenon was also observed in *Ufl1*^*−/−*^ BMDMs (Supplementary Fig. 2E, F). Consistently, *Ufl1*^*−/−*^macrophages contained much more HSV-1 loads and decreased secretion of IFN-β and inflammatory factors (Fig. 2H, I).

### UFL1 regulates cGAS-STING signal pathway

Given that UFMylation modification plays an important role in endoplasmic reticulum homeostasis and ER stress-induced cell death [28, 29], we detected the death of macrophages after UFL1 knocked down. Fluorescence microscope revealed that there was no significant difference with PI staining (Supplementary Fig. 3A). The same results were also obtained by flow cytometry (Supplementary Fig. 3B). Furthermore, there was also no significant difference in lactate dehydrogenase (LDH) release in cell supernatants after HSV-1 infection (Supplementary Fig. 3C). These results suggest that UFL1 does not promote antiviral innate immune response by affecting cell death.

**Fig. 3:**
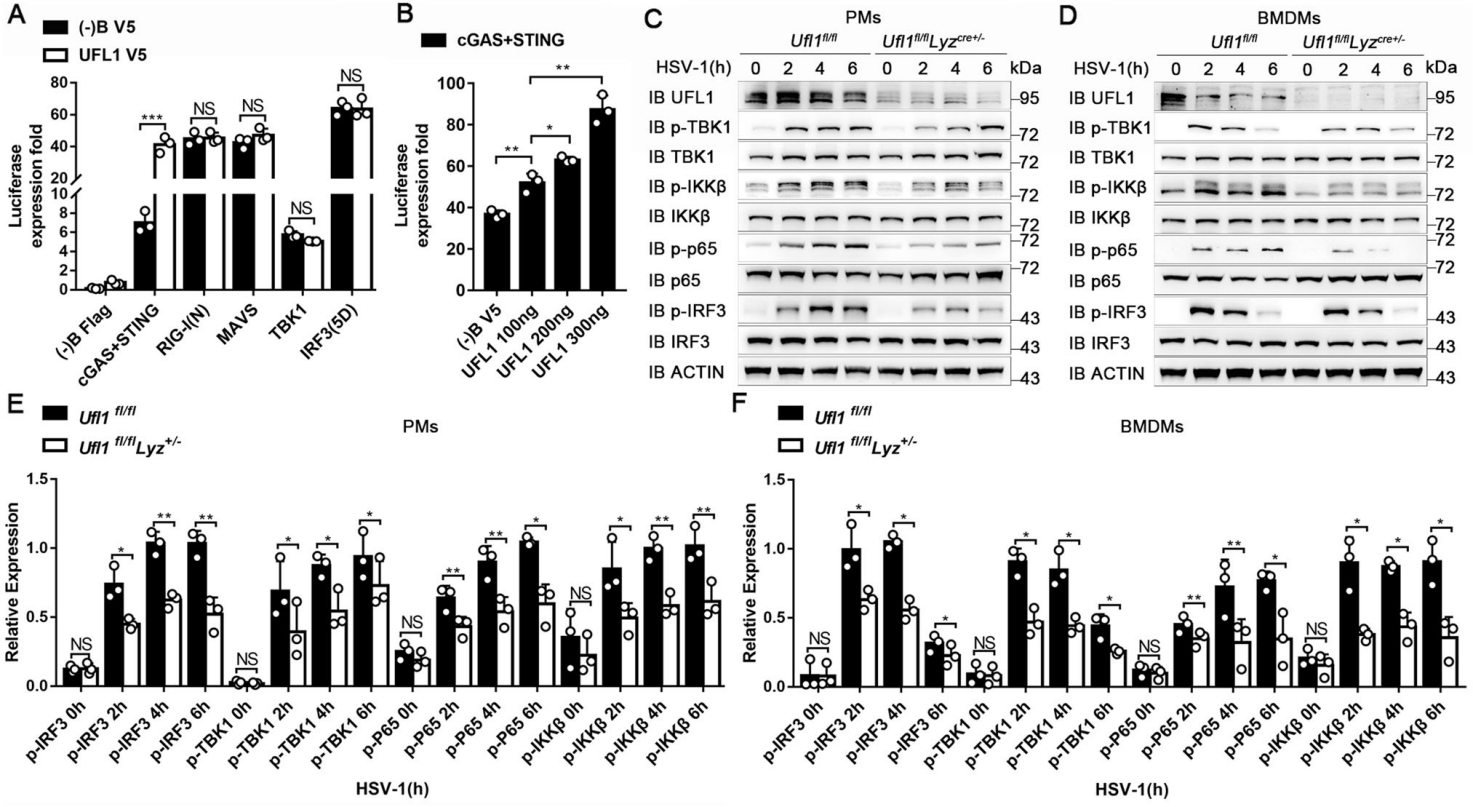
UFL1 targets cGAS-STING signal pathway. **A** IFN-β luciferase activity in HEK293 cells transfected with indicated molecules. **B** cGAS and STING triggered IFN-β luciferase activity in HEK293 cells, transfected with increasing amounts of UFL1. **C**, **D** Phosphorylation of the indicated molecules after HSV-1 infection in PMs (**C**) and BMDMs (**D**) from *Ufl1*^*fl/fl*^ and *Ufl1*^*fl/fl*^*Lyz*^*cre+/***−**^ mice. **E**, **F** Quantification of protein in PMs (**E**) and BMDMs (**F**) via Image J. Data are representative of three independent experiments. *P < 0.05, **P < 0.01, ***P < 0.001.

To elucidate the mechanisms of UFL1 in antiviral innate immunity, luciferase reporter assay was employed to investigate the effect of UFL1 on certain antiviral signal pathways. UFL1 promoted the activation of IFN-β induced by cGAS and STING, but had no effect when triggered by RNA sensor RIG-I, mitochondrial antiviral signaling protein (MAVS) and downstream TBK1, IRF3 (Fig. 3A). More importantly, the effect of UFL1 on cGAS-STING induced IFN-β activation was dose dependent (Fig. 3B). These data strongly indicate that UFL1 interferes with the cGAS-STING pathway, and mainly targets the upstream of TBK1.

Subsequently, the activation of cGAS-STING signal pathway was investigated in *Ufl1-* deficient macrophages. The phosphorylation of TBK1, IRF3, I-kappaB kinase beta (IKKβ) and p65 obviously weakened in *Ufl1*^*−/−*^ PMs (Fig. 3C, E) and BMDMs (Fig. 3D, F) after HSV-1 infection. The same phenomenon was also observed in the livers of *Ufl1*^*−/−*^ mice infected with HSV-1 (Supplementary Fig. 3D). Collectively, these results indicate that deficiency of *Ufl1* significantly inhibits the activation of IRF3 and NF-κB signal pathways, and UFL1 acts on the upstream of TBK1 and IKKβ, namely cGAS or STING.

### UFL1 promotes antiviral innate immunity by targeting STING

**Fig. 4:**
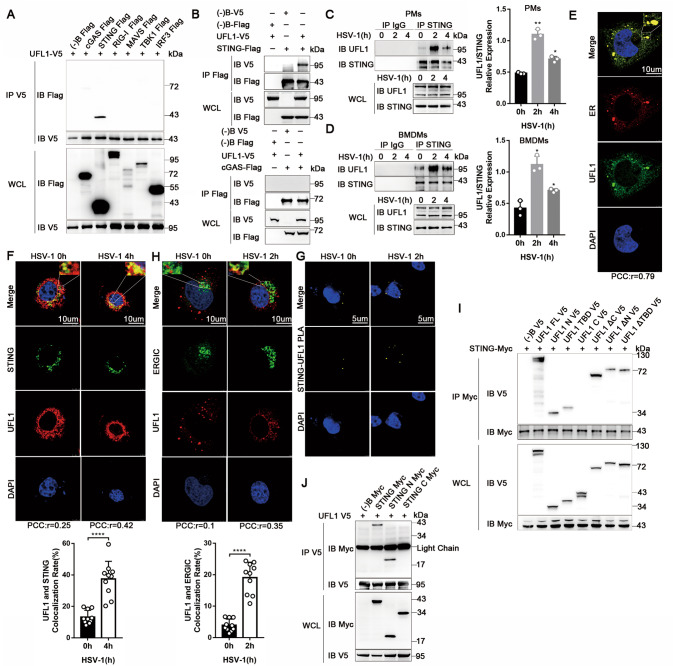
UFL1 interacts with STING. **A** V5-tagged UFL1 and Flag-tagged cGAS, STING, RIG-I, MAVS, TBK1, IRF3 were transfected into HEK293T cells as indicated, and their interaction was tested by V5 immunoprecipitation. **B** Flag-tagged STING or Flag-tagged cGAS were transfected with UFL1 into HEK293T cells, and their interaction was tested by Flag immunoprecipitation. **C**, **D** (Left) Interaction between STING and UFL1 induced by HSV-1 was examined by immunoprecipitation in PMs (**C**) and BMDMs (**D**). (Right) Quantification of UFL1 level normalized by STING in PMs (**C**) and BMDMs (**D**) via Image J. **E** Confocal analysis of endogenous UFL1 and ER co-localization in HELA cells. The bar in the picture stood for 10 µm. The “r” represents PCC value of UFL1 and ER co-localization. **F** (Above) Confocal analysis of UFL1 and STING expression and co-localization in HELA cells stimulated with HSV-1 or not. The bar in the picture stood for 10 µm. (Below) Quantification of the co-localization rate of UFL1 and STING via LAS X software. The “r” represents PCC value of UFL1 and STING co-localization. **G** PLA of STING and UFL1 interaction in 293T cells with HSV-1 stimulated or not. The bar in the picture stood for 5 µm. **H** (Above) Confocal analysis of UFL1 and ERGIC co-localization in HELA cells stimulated with HSV-1 or not. The bar in the picture stood for 10 µm. (Below) Quantification of the co-localization rate of UFL1 and ERGIC via LAS X software. The “r” represents PCC value of UFL1 and ERGIC co-localization. **I** Truncated UFL1 mutants were transfected into HEK293T cells as indicated, and STING-UFL1 interaction was tested by STING immunoprecipitation. **J** Truncated STING mutants were transfected into HEK293T cells as indicated, and UFL1-STING interaction was tested by UFL1 immunoprecipitation. Data shown are representative of three independent experiments with similar results (**A**–**D**, **I**, **J**).

To identify the target of UFL1, key signal molecule of cGAS-STING pathway was cotransfected with UFL1 separately. Co-IP revealed that UFL1 is strongly associated with STING, and may also slightly interact with cGAS (Fig. 4A). Reverse Co-IP assay confirmed that UFL1 interacts with STING, but not cGAS (Fig. 4B). The endogenous combination of UFL1 and STING was also confirmed in the PMs and BMDMs (Fig. 4C, D), and their interaction gradually weakened after HSV-1 infection probably because of STING degradation. UFL1 mainly locates in the endoplasmic reticulum under the unstimulated condition (Fig. 4E and Supplementary Fig. 4A–C) and had been partly co-localized with STING (Fig. 4F). However, in response to HSV-1 stimulation, STING aggregated perinuclearly with enhanced co-localization with UFL1 (Fig. 4F). Proximity ligation assay (PLA) also confirmed that UFL1 can interact with STING in physiologic condition and their interaction enhanced after HSV-1 stimulated (Fig. 4G). STING was previous reported to translocate to endoplasmic reticulum-golgi intermediate compartment (ERGIC) after stimulated [30]. Normally, UFL1 seldom locates in the ERGIC, while their co-localization augmented after HSV-1 stimulated which might be due to UFL1 migrating with STING (Fig. 4H).

To explore the crucial domain for UFL1 and STING interaction, we constructed different truncations of UFL1 (N: N-terminal, TBD: Thyroid hormone receptor interactor4 (TRIP4) binding domain, C: C-terminal, ΔN: TBD and C-terminal, ΔTBD: N-terminal and C-terminal, ΔC: N-terminal and TBD; Supplementary Fig. 4D) and overexpressed STING with full-length (FL) or truncated UFL1 in HEK293T cells followed by Co-IP assay. The results suggest that FL UFL1, together with ΔC truncations, could strongly interact with STING (Fig. 4I and Supplementary Fig. 4E). The interaction greatly reduced with truncations of N, TBD, ΔN, ΔTBD, and truncation C abrogated its binding ability with STING. These results indicate that the N-terminal and TBD domain of UFL1 could both weakly interact with STING, and truncations with both N-terminal and TBD domain robustly increased their interaction.

The N-terminal of STING has four transmembrane domains and the C-terminal is responsible for binding nucleic acids. We constructed N and C terminal truncations of STING respectively (Supplementary Fig. 4F) as well. Only the FL and N-terminal of STING could interact with UFL1 (Fig. 4J). Taken together, the above data indicate that the N-terminal and TBD domain of UFL1 interact with the N-terminal of STING.

### UFL1 promotes antiviral immunity independent of UFMylation

**Fig. 5:**
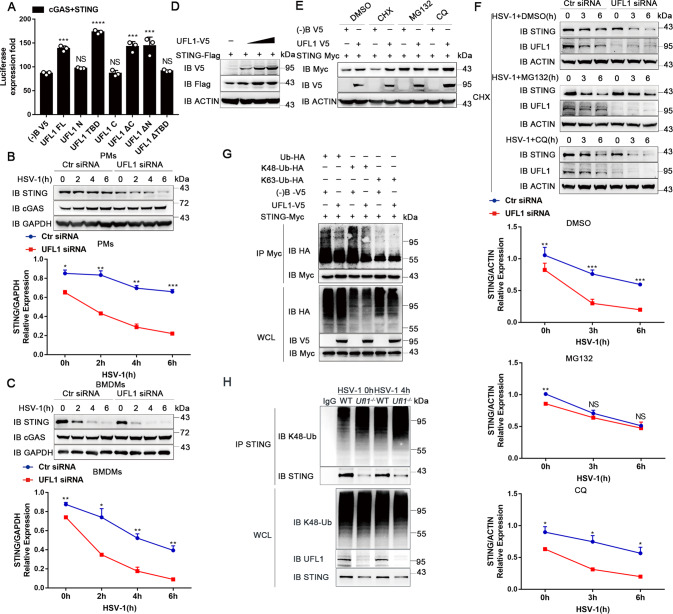
UFL1 inhibits K48-linked ubiquitination and proteasomal degradation of STING. **A** IFN-β luciferase activity in HEK293 cells transfected with cGAS plus STING, and different truncated UFL1 mutants as indicated. Data are presented as means ± SD from three independent experiments. *P < 0.05, **P < 0.01, ***P < 0.001. **B**, **C** (Above) Expression of cGAS and STING post HSV-1 infection in PMs (**B**) and BMDMs (**C**) knockdown of UFL1 or not. (Below) Quantification of STING level normalized by GAPDH via Image J. **D** Expression of STING with increasing doses of V5-tagged UFL1 in MEF cells. **E** Expression of STING in the presence or absence of UFL1, treated with indicated inhibitors. **F** (Above) Expression of STING in PMs with UFL1 knockdown or not, treated with indicated inhibitors. (Below) Quantification of STING level normalized by ACTIN via Image J. **G** The ubiquitination of STING in HEK293T cells transfected with UFL1 and HA-tagged WT or mutant ubiquitin. **H** The endogenous K48-ubiquitination of STING in BMDMs from *Ufl1*^*fl/fl*^ and *Ufl1*^*fl/fl*^*Lyz*^*cre+/***−**^ mice. Data shown are representative of three independent experiments with similar results (**B**–**H**).

Since UFL1 is a specific ligase acts as the E3 to recognize the substrate, we questioned whether UFMylation is involved. Only truncation TBD could fully promote the activation of IFN-β as well as the FL, suggesting that TBD domain of ULF1 is key and enough for its antiviral innate immune response (Fig. 5A). N-terminal of UFL1, where its E3-like ligase activity located, may increase its interaction with STING (Fig. 4H), but is not necessary for the effect of UFL1.

To further determine whether STING can be ufmylated, we coexpressed STING with UFM1 or UFM1^∆C2^ (UFM1 matured only when C-terminal glycine 83 residue was exposed, termed UFM1^∆C2^), along with the necessary components of UFMylation. However, no UFMylation of STING was observed by neither UFM1 nor UFM1^∆C2^ (Supplementary Fig. 5A). In the UFMylation enzymatic reaction, E2 conjugating-enzyme UFC1 is also indispensable as UFL1 [26]. We hypothesized that UFC1 would have the same effect on antiviral innate immune response as UFL1 if UFMylation is involved. To examine our hypothesis, we knocked down UFC1 in peritoneal macrophages (Supplementary Fig. 5B). Decreased expression of UFC1 had no influence on the *Ifnb1* and *Il6* mRNA expression induced by HSV-1 (Supplementary Fig. 5C). Thus, all the above results suggest that UFL1 regulates the antiviral innate immune response independent of UFMylation.

### UFL1 maintains STING stability by inhibiting its proteasomal degradation

Afterward, we investigated the effect of the interaction of UFL1 with STING. Knocking down UFL1 significantly reduced STING expression even without stimulation (Fig. 5B). With the prolongation of infection, the reduced expression of STING was more obvious than control, whereas cGAS was not changed. The mRNA expression of *Sting* was not affected (Supplementary Fig. 5D). The same phenomenon was observed in BMDMs as well (Fig. 5C). In addition, UFL1 dose dependently increased STING expression (Fig. 5D and Supplementary Fig. 5E). These results suggest that UFL1 could specifically increase STING protein expression posttranslationally.

To figure out the specific mechanism of decreased STING expression by UFL1 deficiency, cycloheximide (CHX), proteasome inhibitor MG132 and lysosome inhibitor chloroquinet (CQ) were used to inhibit STING translation or degradation. The increased expression of STING induced by UFL1 was only rescued by MG132 treatment (Fig. 5E and Supplementary Fig. 5F), suggesting UFL1 improved STING expression mainly by inhibiting its proteasomal degradation. MG132 treatment also fully rescued the STING degradation induced by UFL1 knockdown in primary macrophages, while CHX or CQ had no effect (Fig. 5F).

Furthermore, we investigated the ubiquitination of STING in HEK293T cells. Overexpression of UFL1 downregulated total and K48-linked ubiquitination of STING but had no effect on K63-linked ubiquitination (Fig. 5G and Supplementary Fig. 5G). Consistently, endogenous K48-linked ubiquitination of STING was increased in macrophages from *Ufl1*^*−/−*^ mice (Fig. 5H and Supplementary Fig. 5H, I). Collectively, these data suggest that UFL1 suppresses K48-linked ubiquitination and proteasomal degradation of STING.

### UFL1 reduces STING ubiquitination at Lys338/347/370

**Fig. 6:**
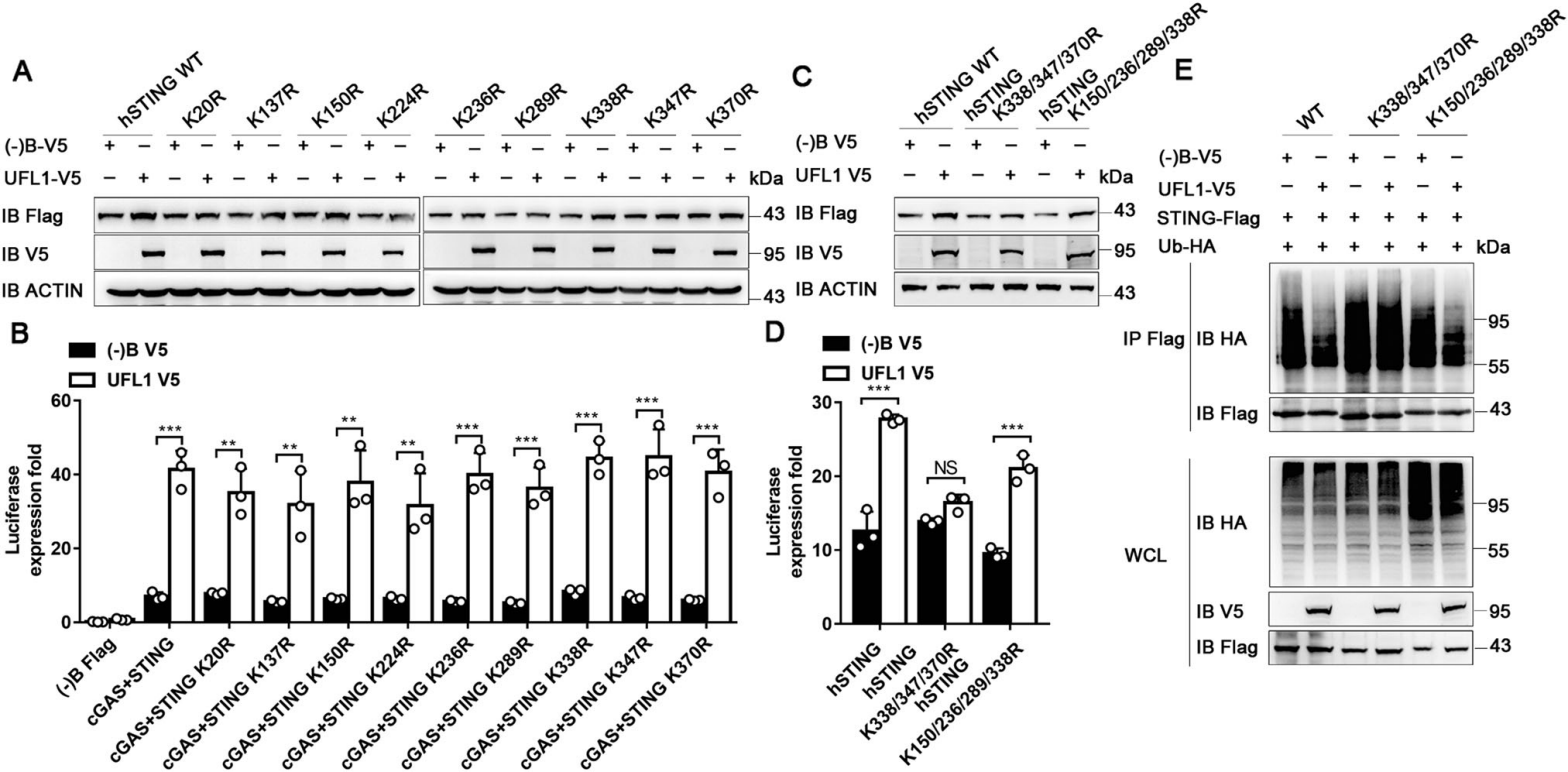
UFL1 reduces STING ubiquitination at Lys338/347/370. **A**, **C** Expression of different single site mutants (**A**) or multiple site mutants (**C**) of STING in HEK293 cells transfected with or without V5-tagged UFL1. **B**, **D** IFN-β luciferase activity in HEK293 cells transfected with different STING mutants as indicated. Data are presented as means ± SD from three independent experiments. *P < 0.05, **P < 0.01, ***P < 0.001. **E** The ubiquitination of different STING mutants in HEK293T cells transfected with V5-tagged UFL1, HA-tagged ubiquitin as indicated. Data shown are representative of three independent experiments with similar results (**A**, **C**, **E**).

To determine the specific STING ubiquitination sites, we replaced each of STING lysine residues individually with arginine. After transfection into HEK293 cells, UFL1 promoted the expression of WT STING and all single mutated STING (K20R, K137R, K150R, K224R, K236R, K289R, K338R, K347R, and K370R) (Fig. 6A and Supplementary Fig. 6A). Accordingly, UFL1 promoted the activation of IFN-β luciferase activity induced by each single lysine residue mutants, as well as WT STING (Fig. 6B). These results suggest that single lysine residue of STING may not enough for the function of UFL1.

Thus, we further constructed STING mutants with multiple lysine sites K150/236/289/338R and K338/347/370R. The expression of WT STING or K150/236/289/338R mutation was strongly enhanced by overexpression of UFL1, but not for K338/347/370R mutation (Fig. 6C and Supplementary Fig. 6B). The luciferase reporter assay also showed that UFL1 could not promote the activation of IFN-β induced by K338/347/370R mutant STING (Fig. 6D). As expected, the ubiquitination of K338/347/370R mutation was not affected by UFL1, while the ubiquitination of WT STING or K150/236/289/338R mutation was significantly weakened by UFL1 (Fig. 6E and Supplementary Fig. 6C). This was further confirmed by K288/337R mutation of mouse STING (Supplementary Fig. 6D, E). All the above results suggest that Lys338, Lys347, and Lys370 of STING are the key sites responsible for the effect of UFL1.

### UFL1 reduces STING ubiquitination by TRIM29

**Fig. 7:**
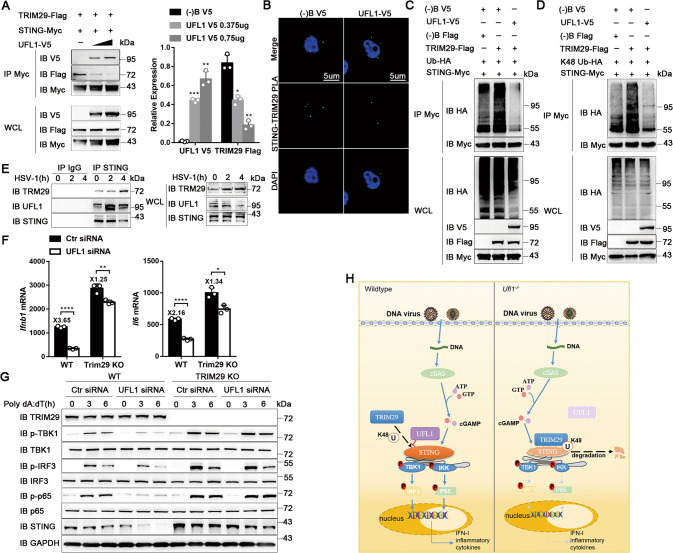
UFL1 reduces STING ubiquitination by competition with TRIM29. **A** (Left) Co-immunoprecipitation and immunoblot analysis of TRIM29-STING and UFL1-STING interaction in 293T cells transfected with varying doses of UFL1. (Right) Quantification of UFL1-V5 and TRIM29-Flag level normalized by STING-Myc via Image J. **B** PLA of STING and TRIM29 interaction in 293T cells with ULF1 overexpressed or not. The bar in the picture stood for 5 µm. **C**, **D** WT (**C**) and K48-linked (**D**) ubiquitination of STING by TRIM29 in HEK293T cells transfected with UFL1 or not. **E** Interaction between UFL1, TRIM29 and STING induced by HSV-1 was examined by immunoprecipitation in BMDMs. **F** *Ifnb* and *Il6* mRNA expression in L929 cells infected with HSV-1. **G** Phosphorylation of the indicated molecules and expression of STING in L929 cells transfected with Poly dA:dT. **H** The mechanism of UFL1 promoting antiviral immune response through reducing STING ubiquitination and degradation. Data shown are representative of three independent experiments with similar results (**A**, **C**–**E**, **G**).

Current studies have showed that E3 ligases RNF5, TRIM30a, and TRIM29 can all regulate the K48-linked ubiquitination of STING to promote its degradation by the proteasome pathway [10–13]. Therefore, we hypothesized that UFL1 might regulate STING ubiqutination indirectly through competing with these E3 ligases. To verify this, we cotransfected TRIM29, RNF5, TRIM30a and STING with different doses of UFL1 and detected their interaction. As the expression of UFL1 increased, its combination with STING enhanced, while the binding of TRIM29 to STING gradually weakened (Fig. 7A). However, UFL1 did not modulate the interaction of RNF5 or TRIM30a with STING (Supplementary Fig. 7A), suggesting that UFL1 selectively competes with TRIM29. PLA also showed the interaction of TRIM29 and STING was decreased with overexpressed UFL1 (Fig. 7B). Additionally, the WT or K48-linked ubiquitination of STING enhanced after TRIM29 overexpression (Fig. 7C, D and Supplementary Fig. 7B, C). However, both of them decreased significantly after transfected with UFL1 (Fig. 7C, D and Supplementary Fig. 7B, C). These results indicate that the competitive interaction between UFL1 and TRIM29 is crucial for STING homeostasis in physiological and viral infected status.

TRIM29 could bind to STING in unstimulated condition, and their interaction enhanced significantly at 4 h after HSV-1 infection, while the interaction between STING and UFL1 peaked at 2 h, and started to weaken at 4 h (Fig. 7E and Supplementary Fig. 7D). Subsequently, we detected whether TRIM29 was indispensable for the effect of UFL1. Without TRIM29, HSV-1 infection induced more production of *Ifnb1* and inflammatory cytokines (Fig. 7F). However, the effect of UFL1 knockdown was greatly abrogated without TRIM29 (Fig. 7F and Supplementary Fig. 7E). *Trim29-*deficiency also increased the STING expression and cGAS-STING pathway activation after Poly dA:dT transfection (Fig. 7G and Supplementary Fig. 7F). However, UFL1 knockdown had no effect on the degradation of STING and signaling activation in *Trim29*^*−/−*^cells (Fig. 7G and Supplementary Fig. 7F). We further detected the UFL1 expression both in WT and *Trim29*^*−/−*^ cells and found it both decreased after infected with HSV-1 (Supplementary Fig. 7G), which means TRIM29 was not responsible for the down-regulation of UFL1. Collectively, these data suggest that UFL1 reduces STING ubiquitination and promotes antiviral innate immunity by competitive with TRIM29 (Fig. 7H).

## Discussion

In this study, we identified UFL1 as a viral infection regulated protein which can promote antiviral innate immunity independent of UFMylation. We found that *Ufl1-*deficiency weakened the antiviral immune response by promoting the degradation of STING and reducing the production of type I interferon. Mechanistically, UFL1 acts as a competitor of STING-TRIM29 interaction and inhibits K48-linked ubiquitination of STING at Lys338/347/370, thereby maintains its stability. The corresponding mechanisms and biological significance of STING ubiquitination may further reveal the entire role of STING in the antiviral innate immunity.

The ER-to-Golgi trafficking is an early rate-limiting process for phosphorylation of STING and activation of downstream signaling pathways [31–34]. After sensing dsDNA, STING would migrate from the ER to the ERGIC, where it is phosphorylated by TBK1 [32], and finally reaches the cytoplasmic punctate structures, the initial location for STING degradation. Therefore, maintaining the early expression and activation of STING is crucial to generate efficient antiviral immune response. In the early stage of HSV-1 infection, the expression of UFL1 (Fig. 1B) and STING (Fig. 5B, C) did not decrease significantly and their interaction peaked at 2 h, suggesting the phosphorylation of STING may promote its interaction with UFL1. This process may be the compensation to the degradative effect of TRIM29 to maintain STING expression during the initial stage of infection. Similar results were obtained by confocal fluorescence experiments. Hardly any co-location between UFL1 and ERGIC was detected in physiologic condition in our study (Fig. 4H). However, UFL1 migrated to ERGIC along with STING after HSV-1 stimulation, which means UFL1 is involved in the early phase activation of the cGAS-STING signaling. Viral infection could dysregulate the UFL1 expression, thus promoted STING degradation by TRIM29 to prevent excessive production of IFN. This strategy may be also employed by the virus, facilitate its amplification in cells. TLRs play a critical role in the recognition of HSV-1 during viral entry and replication. Viral glycoproteins on the envelope, such as gB and gH/gL [35, 36], serve as PAMPs for TLR2. The unmethylated CpG motifs of viral genomic DNA are the only natural ligands for TLR9 [37, 38]. In addition, the viral dsRNA intermediates produced during HSV-1 replication presumably serve as the ligands for TLR3 [39]. Although explicit ligands of HSV-1 for TLR4 and TLR7 are still unclear, both of them were activated during HSV-1 infection [40, 41]. According to our results, the mechanism of UFL1 down-regulation after viral infection might related to TLRs triggered NF-κB pathway.

*Ufl1-*deficiency would reduce the innate immune responses to both DNA and RNA viruses and their underlying mechanisms are different. For DNA virus, UFL1 deficiency would cause the degradation of STING and directly suppress the production of IFN-β, which then leads to increased viral replication. However, while infected with the RNA virus, UFL1 would affect IFN-β indirectly through interfering RNA viral replication. The detailed molecular mechanism for UFL1 in RNA virus replication needs further investigation.

Previous studies of E3-like ligases were mainly focused on their classical functions. Protein inhibitor of activated STAT1 (PIAS1), an E3-like ligase of SUMOylation, was found can target vimentin (VIM) at Lys439 and Lys445 residues and mediate VIM SUMOylation to regulate its dynamic disassembly [42]. HECT and RLD domain containing E3 ubiquitin protein ligase 5 (HERC5) has recently been shown to carry out viral protein ISGylation to promote hepatitis C virus (HCV) proliferation via improved cyclophilin A recruitment by NS5A proteins [43]. In the past few years, UFL1 has been identified as a critical regulator in the context of DNA damage by targeting MRE11 and histone H4 [24, 44]. However, the non-classical functions of E3-like ligases are rarely detected. Our study has identified a novel function of UFL1 that is independent of UFMylation. Although the N-terminal of UFL1, where E3-like ligase activity domain lies in, could bind to STING, it is not indispensable for the function of UFL1 in the reducing of STING ubiquitination and subsequent type I IFN production. What’s more, no UFMylation of STING was observed when coexpressed with full UFMylation components and enzymes. These results proved the role of UFL1 in the regulation of STING stability and antiviral innate immunity is UFMylation-independent. Our research reveals that the traditional E3-like ligase can work independently of the enzymatic cascade, which broadens a new perspective for the effect of UFL1.

The cGAS–STING signal axis is critical to detect pathogenic DNAs to trigger innate immune response against microbial infections. However, host-derived dsDNAs, including extranuclear chromatin resulting from genotoxic stress and mtDNA, can also be recognized by cGAS and produce a potent inflammatory immune response. Under normal conditions, leakage of mtDNA occurs when mitochondrial outer membrane permeabilization in the context of intrinsic apoptosis [45]. However, it has been proved that microbial pathogens can induce mitochondrial stress and cause mtDNA leakage to trigger cGAS-STING activation. Mycobacterium tuberculosis can trigger STING through mitochondrial dynamics and mtDNA to contribute to IFN-β induction [46, 47]. Therefore, UFL1 may be a broad target for immune therapy. On the other hand, overactivation of cGAS-STING signal may cause severe inflammatory pathologies including autoinflammation and autoimmunity. Studies have illustrated that Aicardi-Goutières syndrome (AGS) and systemic lupus erythematosus (SLE) are all resulted from systemic activation of cGAS–STING signal axis [48, 49]. Our confocal assay proved that UFL1 interacts with STING under the unstimulated condition which means the combination of UFL1 and STING is constitutive, it may provide a new therapeutic target for inhibiting autoimmune disease triggered by abnormally activated STING.

In summary, our findings illustrated the crucial role of UFL1 in antiviral innate immune response. UFL1 is downregulated by DNA virus infection and mediates a positive regulation of cGAS-STING signal pathway for the production of type I interferon and proinflammatory cytokines. Our study reveals a novel non-classical mechanism that the E3-like ligase UFL1 can maintain the expression of STING independent of UFMylation, which provides new strategy to improve antiviral therapy and to regulate self-DNA triggered autoimmunity.

## Methods

### Mice

*Ufl1*^*fl/fl*^ mouse was constructed by Cyagen Biotech (Suzhou, China) using CRISPR/Cas9 techniques and then mated with *Lyz-cre* transgenic mouse, obtained from The Jackson Laboratory (Bar Harbor, ME), to generate myeloid-specific deficiency. C57BL/6 J mice were purchased from Joint Ventures Sipper BK Experimental Animals (Shanghai, China). Mice were kept and bred in specific-pathogen-free conditions. All animal experiments were undertaken in accordance with the National Institute of Health Guide for the Care and Use of Laboratory Animals with approval of the Scientific Investigation Board of Naval Medical University, Shanghai.

### Cell culture

Peritoneal macrophages were harvested from mice four days after thioglycollate (BD, Sparks, MD) injection. Bone marrow cells were cultured with recombinant mouse M-CSF (20 ng/ml) for generation of BMDM. The BHK21, HEK293T, HEK293, MEF, A549, L929 and HELA cell lines were obtained from the American Type Culture Collection. All cells were cultured in DMEM (Gibco) supplemented with 10% FCS (Gibco) in a 5% CO_2_ atmosphere at 37 °C.

### Viruses and virologic assays

HSV-1 virus (Kos strain, kindly provided by Dr. Qihan Li, Chinese Academy of Medical Sciences, China) was obtained as indicated. Viral titers of stocks and experimental samples were determined by TCID50 on BHK21 cells.

### Quantitative Real-Time RT-PCR

Total RNA was extracted from cultured cells with TRIzol reagent according to the manufacturer’s instructions. RNA was reverse transcribed with Oligo (dT) primer for mRNA into cDNA with M-MLV Reverse Transcriptase (TaKaRa). RNA expression was quantified by real-time PCR with SYBR Premix ExTaq Kit (TaKaRa) and normalized by the level of β-actin. A 2^−∆∆Ct^ method was used to calculate relative expression changes. Amplification of cDNA was performed on the ABI-Quant Studio 7 Flex. The sequences of the primers for quantitative real-time RT-PCR are listed in Key resources table (Supplementary information).

### Immunoprecipitation and Western blot

Cells or tissues were harvested and lysed with cell lysis buffer (Cell Signaling Technology) supplemented with protease inhibitor cocktail (Roche). Protein concentrations of the lysates were measured with bicinchoninic acid (BCA) assay (Pierce) and equalized with the lysis buffer. Equal amount of the extracts was used for immunoprecipitation and immunoblot analysis. Protein levels were quantified using Image J software and normalized to the internal control β-actin or Gapdh.

### RNA interference

Mouse peritoneal macrophages, bone marrow derived macrophage, L929 cells and A549 cells were transfected with siRNA (20 nM) through the use of Lipofectamine® RNAiMAX Reagent (Invitrogen). The mouse specific siRNA targeting *Ufl1* and *Ufc1*, human specific siRNA targeting *UFL1* were designed and synthesized by GenePharma Co (Shanghai, China) and the siRNA targeted sequence are listed in Key resources table (Supplementary information).

### Virus Infection

Cells were infected with HSV-1 or VACV for the indicated hours. Related genes and viral RNA expression were detected 6 h later and cytokine production was analyzed 24 h later. For in vivo cytokine production studies, age- and sex-matched groups of littermate mice were i.v. infected with HSV-1 (1 × 10^8^ plaque-forming units per gram body weight).

### ELISA

Secreted cytokines in cell culture supernatants or serum from virus infected-mice were analyzed using mouse IFN-β (Biolegend), mouse IL-6 and TNF (R&D Systems) ELISA kits according to the manufacturer’s instructions.

### LDH assay

Cells were plated at 5 × 10^5^ cells per ml in 24-well plates (500 μl per well) and treated as required. LDH release was assayed using the Cytotoxicity Assay kit (Beyotime Biotechnology) according to the manufacturer’s instructions.

### Flow cytometry

Immune cell populations: The phenotypes and proportions of neutrophils, macrophages and dendritic cells in the spleen of *Ufl1*^*fl/fl*^ and *Ufl1* ^*fl/fl*^*Lyz*^*cre+/***−**^ mice were determined by flow cytometry. Data were obtained on Fortessa (BD Bioscience). Apoptosis: Cells were plated at 5 × 10^5^ cells per ml in 24-well plates (500 μl per well) and treated as required. At the end of the stimulation, cells were harvested and washed with PBS. After treated with propidium iodide for 15 min, cells were transferred to polypropylene FACS tubes. Data were obtained on LSR II (BD Bioscience) and analyzed using FlowJo software.

### Immunofluorescence and Confocal Microscopy

Cells were plated at 2 × 10^5^ cells per ml in 6-well plates (2 ml per well) and treated as required. Cells were fixed in PBS containing 4% formaldehyde, permeabilized with 0.2% saporin with 5% BSA and 10% FCS, and then stained with indicated antibody. Images were obtained with laser scanning confocal microscope (Leica TCS SP8). The co-localization rate is analyzed by the LAS X software version 2.0.2.15022. Pearson co-localization coefficient (r represents PCC value) is analyzed by the Fiji software. The value of r ranging from 0.00–0.20 is regarded as “very weak”, >0.20–0.40 as “weak”, >0.40–0.60 as “moderate”, >0.60–0.80 as “strong”, and > 0.80–1.0 as “very strong”.

### Proximity ligation assay

The exogenous interaction of STING and UFL1/TRIM29 was detected in HEK293T cells cotransfected with STING-Myc and UFL1-V5/TRIM29-Flag plasmids. PLA was performed with Duolink (Sigma-Aldrich, USA) In Situ PLA technology-related kits, including In Situ Detection Reagents Red (DUO92008), In Situ PLA Probe Anti-Rabbit PLUS (DUO92002) and In Situ PLA Probe Anti-Mouse MINUS (DUO92004). All incubation, ligation and amplification procedures were done according to manufacturer’s instructions.

### Statistical Analysis

Statistical significance was assessed by unpaired two-tailed Student’s *t* test with a value of *p* < 0.05 considered to be statistically significant (*p < 0.05, **p < 0.01, ***p < 0.001, NS, not significant.). The statistical tests were justified as appropriate according to assessment of normality and variance of the distribution of the data. No randomization or exclusion of data points was used. No ‘blinding’ of investigators was applied.

## Supplementary information

Reproducibility Checklist

Author contribution form

Supplementary Tables

Supplementary information

Supplementary Figure 1

Supplementary Figure 2

Supplementary Figure 3

Supplementary Figure 4

Supplementary Figure 5

Supplementary Figure 6

Supplementary Figure 7

Supplemental Material-western blots

## Data Availability

Data supporting the present study are available from the corresponding author upon reasonable request.
