# Supplementary material for: UFL1 promotes antiviral immune response by maintaining STING stability independent of UFMylation
Source: Cell Death Differ. 2022 Jul 23;30(1):16–26. doi: 10.1038/s41418-022-01041-9 (PMC9883236; doi:10.1038/s41418-022-01041-9)
Supplement: Supplementary file 2 — Author contribution form [file 41418_2022_1041_MOESM2_ESM.pdf]

DECLARATION OF CONTRIBUTIONS TO ARTICLE

ADMC

Manuscript Number: CDD-22-0101R

Journal Name: *Cell Death & Differentiation* (the 'Journal')

Proposed Title of the Contribution:

UFL1 Promotes Antiviral Immune Response by Maintaining STING Stability Independent of UFMylation. (the 'Contribution)

Author(s):

Yijie Tao, Shulei Yin, Yang Liu, Chunzhen Li, Yining Chen, Dan Han, Jingyi Huang, Sheng Xu, Zui Zou ,Yizhi Yu (the 'Authors')

For all *CDD* articles, each person named as an author in the published version must be able to show he or she has contributed substantially to the article.

Authorship credit should be based on 1) substantial contributions to conception and design, acquisition of data, or analysis and interpretation of data; 2) drafting the article or revising it critically for important intellectual content; and 3) final approval of the version to be published. Authors should meet conditions 1, 2 and 3.

Any person who cannot be shown to have made a substantial contribution to the article cannot be listed as an author in the final version. The name of any person who is deemed to have made a minor contribution can, however, appear in the Acknowledgments section of the article.

Please complete the table below to indicate the contributions of all named authors to the manuscript.

Author Full Name:	Specification of Contribution to the Manuscript:
Yijie Tao	YT performed experiments, analyzed data, wrote and revised the manuscript.
Shulei Yin	SY performed experiments, analyzed data.
Yang Liu	YL performed experiments, analyzed data.
Chunzhen Li	CL helped to perform experiments, data analysis.
Yining Chen	YC helped to perform experiments, data analysis.
Dan Han	DH helped to perform experiments, data analysis and undertook project management.
Jingyi Huang	JH helped to perform experiments, data analysis and undertook project management.
Sheng Xu	SX designed the experiments,interpreted data and reviewed the manuscript.
Zui Zou	ZZ designed the experiments,interpreted data and reviewed the manuscript.
Yizhi Yu	YY designed the experiments,interpreted data,reviewed the manuscript and approved publishment.

Please complete the table below to indicate the contributions of all named authors to the figures.

Figure 1:

A-F, H, I YT and CL performed the q-PCR and western blot, generated graphs and figures.
G SY and YC performed the q-PCR and generated graphs.
SX, YT and SY analyzed the data in Fig 1.

Figure 2:

A-E YL and DH generated the Ufl1-/- mice and performed the experiments in vivo.
F-I YT and JH performed the q-PCR and generated graphs.
ZZ, YL and YT analyzed the data in Fig 2.

Figure 3:

A-D SY and CL performed the luciferase reporter assay and western blot, generated graphs and figures.
E, F SY and CL analyzed the data by image J and generated graphs.
SX and SY analyzed the data in Fig 3.

Figure 4:

A-D, I, J YT and YC performed the immunoprecipitation assay and western blot, generated graphs and figures.
E-H SY and DH performed the confocal assay and YT performed the PLA assay.
YY, SX, YT and SY analyzed the data in Fig 4.

Figure 5:

A-G YT and CL performed the luciferase reporter assay and western blot, generated graphs and figures. YT and YC analyzed the data by image J and generated graphs.
H YL and YC performed the western blot and generated figures.
YY, ZZ, YT and YL analyzed the data in Fig 5.

Figure 6:

A-E YL and SY constructed the mutants, performed the luciferase reporter assay and western blot, and generated graphs and figures.
ZZ, YY, SY and YL analyzed the data in Fig 6.

Signed for and on behalf of the Author(s):	Print Name:	Date:
Yizhi Yu Zui Zou Sheng Xu	Yizhi Yu;Zui Zou;Sheng Xu	June 28th,2022

Please complete the table below to indicate the contributions of all named authors to the figures.

Figure 7:

A, C-E SY and YT performed the immunoprecipitation assay and western blot.
B YT and DH performed the PLA assay.
F, G YT and SY generated the Trim29 KO cells and performed the experiments.
YY, SX, SY and YT analyzed the data in Fig 7.

Supplementary Figure 1:

A YT and DH analyzed the data by image J and generated graphs.
B-M YT and SY performed the q-PCR and generated graphs.
SX, YT and SY analyzed the data in Supplementary Fig 1.

Supplementary Figure 2:

B YL and JH weighted the mice and generated graphs.
C-F YT and CL performed the flow cytometry analysis and q-PCR.
ZZ, YL and YT analyzed the data in Supplementary Fig 2.

Supplementary Figure 3:

A, D SY and YC performed the live cell imaging under fluorescence microscope and western blot.
B, C YT and CL performed the flow cytometry analysis and LDH assay.
SX, SY and YT analyzed the data in Supplementary Fig 3.

Supplementary Figure 4:

A-C SY and DH performed the confocal assay and generated graphs.
D, F YT and JH constructed different truncations.
E YT and JH analyzed the data by image J and generated graphs.
YY, SX, SY and YT analyzed the data in Supplementary Fig 4.

Supplementary Figure 5:

A-C, I YT and CL performed the western blot and q-PCR, generated graphs and figures.
E-H YT and DH analyzed the data by image J and generated graphs.
YY, ZZ and YT analyzed the data in Supplementary Fig 5.

Signed for and on behalf of the Author(s):	Print Name:	Date:
Yizhi Yu zui zou sheng xu	Yizhi Yu;Zui Zou;Sheng Xu	June 28th,2022

Please complete the table below to indicate the contributions of all named authors to the figures.

Supplementary Figure 6:

A-C YL and JH analyzed the data by image J and generated graphs.
D, E YS and YC performed the luciferase reporter assay and western blot, generated graphs and figures.
ZZ, YY, SY and YL analyzed the data in Supplementary Fig 6.

Supplementary Figure 7:

A YT and CL performed the immunoprecipitation assay and western blot, generated graphs and figures.
B-D SY and CL analyzed the data by image J and generated graphs.
E, G YT performed the q-PCR and generated graphs.
F SY and YC analyzed the data by image J and generated graphs.
YY, YT and SY analyzed the data in Supplementary Fig 7.

Signed for and on behalf of the Author(s):	Print Name:	Date:
Yizhi Yu Zui Zou Sheng Xu	Yizhi Yu;Zui Zou;Sheng Xu	June 28th,2022
