# Supplementary material for: UFL1 promotes antiviral immune response by maintaining STING stability independent of UFMylation
Source: Cell Death Differ. 2022 Jul 23;30(1):16–26. doi: 10.1038/s41418-022-01041-9 (PMC9883236; doi:10.1038/s41418-022-01041-9)
Supplement: Supplementary file 3 — Supplementary Tables [file 41418_2022_1041_MOESM3_ESM.docx]

**Supplementary Tables**

**Key resources table**

| REAGENT or RESOURCE | SOURCE | IDENTIFIER |
| --- | --- | --- |
| Antibodies | | |
| anti-cGAS | Cell Signaling Technology | Cat# 13647 |
| anti-STING | Cell Signaling Technology | Cat# 31659 |
| anti-TBK1 (D1B4) | Cell Signaling Technology | Cat# 3504 |
| anti-p-TBK1 | Cell Signaling Technology | Cat# 5483 |
| anti-IRF-3 (D83B9) | Cell Signaling Technology | Cat# 4302 |
| anti-p-IRF-3 (Ser396) | Cell Signaling Technology | Cat# 4947 |
| anti-IKKβ | Cell Signaling Technology | Cat# 2678 |
| anti-p-IKKβ | Cell Signaling Technology | Cat# 3697 |
| anti-UFL1 | Aviva Systems Biology | Cat#ARP552288-P050 |
| anti-TRIM29 | Santa Cruz | Cat#SC-376125 |
| anti-GAPDH (14C10) | Cell Signaling Technology | Cat# 2118 |
| anti-β-Actin (13E5) | Cell Signaling Technology | Cat# 4970 |
| Bacterial and Virus Strains | | |
| herpes simplex virus (HSV-1) | provided by Dr. Qihan Li | N/A |
| Biological Sample | | |
| Chemicals, Peptides, and Recombinant Proteins | | |
| Lipofectamine™ RNAiMAX | Invitrogen | Cat# 13778 |
| jetPEI | polyplus | Cat#101-40N |
| Trizol | Invitrogen | Cat# 15596018 |
| Recombinant Murine M-CSF | PeproTech | Cat# 315-02 |
| poly dA:dT | Invivogen | Cat# tlrl-patn |
| poly dG:dC | Invivogen | Cat# tlrl-pgcn |
| ISD | Invivogen | Cat# tlrl-isdn |
| cGAMP | Invivogen | Cat# tlrl-nacga23-02 |
| Cycloheximide | MedChemExpress | Cat#HY-12320 |
| MG132 | MedChemExpress | Cat#HY-13259 |
| Chloroquine | MedChemExpress | Cat#HY-17589A |
| Pam3CSK4 | Invivogen | Cat#tlrl-pms |
| Poly I:C | Invivogen | Cat#tlrl-picw-250 |
| LPS | Invivogen | Cat#tlrl-rslps |
| BAY11-7082 | Beyotime Biotech | SF0011 |
| U0126 | Beyotime Biotech | S1901 |
| SP600125 | MedChemExpress | HY-12041 |
| SB239063 | MedChemExpress | HY-11068 |
| SCH772984 | MedChemExpress | HY-50846 |
| Critical Commercial Assays | | |
| LDH Cytotoxicity Assay Kit | Beyotime Biotech | Cat# C0016 |
| LEGEND MAXTM Mouse IFN-β ELISA Kit | Biolegend | Cat# 439408 |
| Mouse IL-6 Quantikine ELISA Kit | R&D systems | Cat# SM6000B |
| Mouse TNF-alpha Quantikine ELISA Kit | R&D systems | Cat# SMTA00B |
| Experimental Models: Cell Lines | | |
| BHK21 | ATCC | Cat # CCL-10 |
| MEF | ATCC | Cat# SCRC-1008 |
| HEK 293T | ATCC | Cat# CRL-11268 |
| HEK 293 | ATCC | Cat# CRL-1573 |
| HELA | ATCC | Cat# CCL-2 |
| L929 | ATCC | Cat# CCL-1 |
| A549 | ATCC | Cat# CCL-185 |
| Experimental Models: Organisms/Strains | | |
| C57BL/6 mice | Shanghai Laboratory Animal Center, Chinese Academy of Science | N/A |
| *Ufl1*^-/-^ mice | This paper | N/A |
| *Irf3* deficient (*Irf3*^-/-^)mice | This paper | N/A |
| Oligonucleotides | | |
| siRNA: mouse *Ufl1* sense (5’-3’):  CAGAUGGUACUGGGACAATT | Genepharma http://www.genepharma.com/ | N/A |
| siRNA: human *Ufl1* sense (5’-3’):  GUGGUCGAGUAAACAUUGUTT | Genepharma | N/A |
| siRNA: mouse *Ufc1* sense (5’-3’):  GCCUAAGUUUGGACUAGCUTT | Genepharma | N/A |
| Primer: mouse *β-actin* (FWD):  AGTGTGACGTTGACATCCGT | This paper | N/A |
| Primer: mouse *β-actin* (REV):  GCAGCTCAGTAACAGTCCGC | This paper | N/A |
| Primer: mouse *Ufl1* (FWD):  AGTCCACGCAAAGGTTGTCAG | This paper | N/A |
| Primer: mouse *Ufl1* (REV):  TTCCTTGCCGTCAAGTGTATG | This paper | N/A |
| Primer: mouse *Ifnb1* (FWD):  CCCTATGGAGATGACGGAGA | This paper | N/A |
| Primer: mouse *Ifnb1* (REV):  TCCCACGTCAATCTTTCCTC | This paper | N/A |
| Primer: mouse *Tnf* (FWD):  GCCTCTTCTCATTCCTGCTT | This paper | N/A |
| Primer: mouse *Tnf* (REV):  TGGGAACTTCTCATCCCTTTG | This paper | N/A |
| Primer: mouse *Il6* (FWD):  CCGGAGAGGAGACTTCACAG | This paper | N/A |
| Primer: mouse *Il6* (REV):  TCCACGATTTCCCAGAGAAC | This paper | N/A |
| Primer: HSV TK (FWD):  AGTTGCGTGGTGGTGGTTT | This paper | N/A |
| Primer: HSV TK (REV):  ACAAAAAGCCACGGAAGTCC | This paper | N/A |
| Primer: mouse *Ufc1* (FWD):  CGGGTCGTGTCTGAGATCC | This paper | N/A |
| Primer: mouse *Ufc1* (REV):  GGTCCCTTCCTTGTTGGACT | This paper | N/A |
| Primer: mouse *Sting* (FWD):  GGTCACCGCTCCAAATATGTAG | This paper | N/A |
| Primer: mouse *Sting* (REV):  CAGTAGTCCAAGTTCGTGCGA | This paper | N/A |
| Primer: human *Ufl1* (FWD):  GCCTGGGAAGAGATTAGGCG | This paper | N/A |
| Primer: human *Ufl1* (REV):  CAACAATGTTTACTCGACCACCT | This paper | N/A |
| Primer: human *Ifnb1* (FWD):  GCTTGGATTCCTACAAAGAAGCA | This paper | N/A |
| Primer: human *Ifnb1* (REV):  ATAGATGGTCAATGCGGCGTC | This paper | N/A |
| Primer: human *Il6* (FWD):  ACTCACCTCTTCAGAACGAATTG | This paper | N/A |
| Primer: human *Il6* (REV):  CCATCTTTGGAAGGTTCAGGTTG | This paper | N/A |
| Software and Algorithms | | |
| Leica laser scanning software | Leica | http://www.leica-microsystems.com/products/confocal-microscopes/details/product/leica-tcs-sp8/ |
| Flowjo_V10 | FlowJo | https://www.flowjo.com/ |
| Image J | Image J | https://imagej.nih.gov/ij/download.html |
| GraphPad.Prism.7.0.4 | GraphPad | https://www.graphpad.com/demos |
