# Supplementary material for: UFL1 promotes antiviral immune response by maintaining STING stability independent of UFMylation
Source: Cell Death Differ. 2022 Jul 23;30(1):16–26. doi: 10.1038/s41418-022-01041-9 (PMC9883236; doi:10.1038/s41418-022-01041-9)
Supplement: Supplementary file 4 — Supplementary information [file 41418_2022_1041_MOESM4_ESM.docx]

**Supplementary information**

UFL1 Promotes Antiviral Immune Response by Maintaining STING Stability Independent of UFMylation

Yijie Tao^1, 4^, Shulei Yin^1, 4^, Yang Liu^2, 4^, Chunzhen Li^1^, Yining Chen^1^, Dan Han^1^, Jingyi Huang^1^, Sheng Xu^1*^, Zui Zou^1, 2, 3*^, Yizhi Yu^1*^.

^1^National Key Laboratory of Medical Immunology and Institute of Immunology, Naval Medical University, Shanghai 200433, China.

^2^Department of Anesthesiology, Changzheng Hospital, Second Affiliated Hospital of Naval Medical University, Shanghai 200003, China.

^3^School of Anesthesiology, Naval Medical University, 168 Changhai Road, Shanghai 200433, China.

^4^These authors contributed equally to this work: Yijie Tao, Shulei Yin, Yang Liu.

*Corresponding author Email: xusheng@immunol.org; zouzui@smmu.edu.cn; yuyz@immunol.org.

**Supplementary figure legends**

**Supplementary Fig. 1 UFL1 promotes antiviral innate immunity.**

**A** Quantification of UFL1 level normalized by GAPDH or ACTIN via Image J. **B** *Ufl1* expression in *Irf3*^-/-^ PMs infected with HSV-1 by qRT-PCR. **C** *Ufl1* expression in *cgas*^-/-^ L929 cells infected with HSV-1 by qRT-PCR. **D** *Ufl1* expression in PMs stimulated with different ligands for TLRs (TLR2: Pam3CSK4, TLR3: Poly I:C, TLR4: LPS). **E** *Ufl1* expression in PMs treated with indicated inhibitors (NF-κB: BAY-117082, MEK: U0126, JNK: SP600125, p38: SB239063, ERK: SCH772984) and infected with HSV-1 by qRT-PCR. **F, K** *Ufl1* expression in PMs (**F**), *Irf3*^-/-^ PMs (**K**) transfected with Ctr-siRNA or UFL1-siRNA for 48 hr. **G** *Ifnb1*, *Il6*, or *Tnf* mRNA expression in BMDMs infected with HSV-1. **H** *UFL1* mRNA expression in A549 cells transfected with Ctr-siRNA or UFL1-siRNA for 48 hr. **I, J** HSV-1 TK RNA expression in PMs (**I**) and BMDMs (**J**) transfected with Ctr-siRNA or UFL1-siRNA and infected with HSV-1. **L, M** VSV RNA expression in PMs (**L**) and *Irf3*^-/-^ PMs (**M**) transfected with Ctr-siRNA or UFL1-siRNA and infected with VSV. Data are presented as means ± SD from three independent experiments. *P < 0.05, **P < 0.01, ***P < 0.001.

**Supplementary Fig.2 *Ufl1* deficiency suppresses IFN-β and inflammatory cytokines production in macrophages.**

**A** Construction of *Ufl1^fl/fl^* mice. **B** Body weight (left) and spleen weight (right) analysis of 8-week-old female *Ufl1^fl/fl^* and *Ufl1^fl/fl^Lyz^cre+/^*^-^ mice. **C, D** Flow cytometry analysis of neutrophils, dendritic cells and macrophages in spleen of 8-week-old female *Ufl1^fl/fl^* and *Ufl1^fl/fl^Lyz^cre+/^*^-^ mice. **E** *Ifnb1*, *Il6*, or *Tnf* mRNA expression in BMDMs from *Ufl1^fl/fl^* and *Ufl1^fl/fl^Lyz^cre+/^*^-^ mice infected with HSV-1. **F** *Ifnb1*, *Il6*, or *Tnf* mRNA expression in BMDMs from *Ufl1^fl/fl^* and *Ufl1^fl/fl^Lyz^cre+/^*^-^ mice transfected with cGAMP and Poly dG:dC. Data are presented as means ± SD from three independent experiments. *P < 0.05, **P < 0.01, ***P < 0.001.

**Supplementary Fig.3 UFL1 has no influence on cell death after HSV-1 infection.**

**A** PMs with UFL1 knockdown or not were infected with HSV-1 and stained with PI. Cells were viewed under fluorescence microscope. **B** PMs with UFL1 knockdown or not were infected with HSV-1 for 12hr and analyzed by FACS LSR II. **C** Determination of LDH in supernatants of PMs with UFL1 knockdown or not and infected with HSV-1 for 12hr. **D** (Left) Phosphorylation of the indicated molecules in the livers from *Ufl1^fl/fl^* and *Ufl1^fl/fl^Lyz^cre+/^*^-^ mice in response to i.v. infection with HSV-1. (Right) Quantification of protein with Image J. Data are representative of three independent experiments. *P < 0.05, **P < 0.01, ***P < 0.001.

**Supplementary Fig.4 UFL1 interacts with STING.**

**A, B** (Left) Confocal analysis of endogenous UFL1 expression and co-localization with ER in PMs (**A**) and BMDMs (**B**). The bar in the picture stood for 10um and 5um. Pearson co-localization coefficient of UFL1 and ER co-localization in PMs or BMDMs is analyzed by the Fiji software. The “ r ” represents PCC value of UFL1 and ER co-localization. **C** Quantification of the co-localization rate of UFL1 and ER in HELA cells, PMs and BMDMs. **D, F** Schematic structure and truncates of UFL1 (**D**) and STING (**F**). **E** Quantification of UFL1-V5 level normalized by STING-Myc via Image J. Data are representative of three independent experiments. *P < 0.05, **P < 0.01, ***P < 0.001.

**Supplementary Fig.5 UFL1 promotes antiviral immunity independent of UFMylation.**

**A** The UFMylation of STING in HEK293T cells. **B** *Ufc1* expression in PMs transfected with Ctr-siRNA or UFC1-siRNA for 48 hr. **C** *Ifnb1*, *Il6*, or *Tnf* mRNA expression in PMs infected with HSV-1. **D** *Sting* expression in PMs transfected with Ctr-siRNA or UFL1-siRNA for 48 hr. **E** Quantification of STING level normalized by ACTIN via Image J. **F** Quantification of STING-Myc level normalized by ACTIN via Image J. **G** Quantification of HA level normalized by STING-Flag via Image J. **H** Quantification of K48-Ub level normalized by STING in BMDMs from *Ufl1^fl/fl^* and *Ufl1^fl/fl^Lyz^cre+/^*^-^ via Image J. **I** (Left) The endogenous K48-ubiquitination of STING in PMs from *Ufl1^fl/fl^* and *Ufl1^fl/fl^Lyz^cre+/^*^-^ mice. (Right) Quantification of K48-Ub level normalized by STING in PMs from *Ufl1^fl/fl^* and *Ufl1^fl/fl^Lyz^cre+/^*^-^ via Image J. Data are presented as means ± SD from three independent experiments. *P < 0.05, **P < 0.01, ***P < 0.001.

**Supplementary Fig. 6** **UFL1 reduces STING ubiquitination at multiple lysine residues.**

**A, B** Quantification of different single site mutants (**A**) or multiple site mutants (**B**) of STING level normalized by ACTIN via Image J. **C** Quantification of HA level normalized by multiple site mutants of STING-Flag via Image J. **D** (Left) Expression of STING in HEK293 cells transfected with V5-tagged UFL1, Flag- tagged STING and multiple mouse STING mutants as indicated. (Right) Quantification of multiple site mutants of mSTING-Flag level normalized by ACTIN via Image J. **E** STING induced IFN-β luciferase activity in HEK293 cells transfected with different mouse STING mutants with or without UFL1. The results are relative to Renilla luciferase activity. Data are presented as means ± SD from three independent experiments. *P < 0.05, **P < 0.01, ***P < 0.001.

**Supplementary Fig. 7 UFL1 reduces STING ubiquitination by competition with TRIM29.**

**A** (Above) Co-immunoprecipitation and immunoblot analysis of RNF5-STING and TRIM30a-STING interaction in HEK293T cells transfected with or without UFL1. (Below) Quantification of UFL1-V5, RNF5-Myc and TRIM30a-Myc level normalized by STING-Flag via Image J. **B, C** Quantification of WT-Ub HA (**B**) or K48-Ub HA (**C**) level normalized by STING-Myc via Image J. **D** Quantification of UFL1 and TRIM29 level normalized by STING in BMDMs via Image J. **E** *Ufl1* expression in L929 cells transfected with Ctr-siRNA or UFL1-siRNA for 48 hr. **F** Quantification of protein in L929 cells after Poly dA:dT transfection via Image J. **G** *Ufl1* expression in WT and Trim29 KO cells infected with HSV-1. Data are presented as means ± SD from three independent experiments. *P < 0.05, **P < 0.01, ***P < 0.001.
