# Supplementary figures and images for: UFL1 promotes antiviral immune response by maintaining STING stability independent of UFMylation
Source: Cell Death Differ. 2022 Jul 23;30(1):16–26. doi: 10.1038/s41418-022-01041-9 (PMC9883236; doi:10.1038/s41418-022-01041-9)

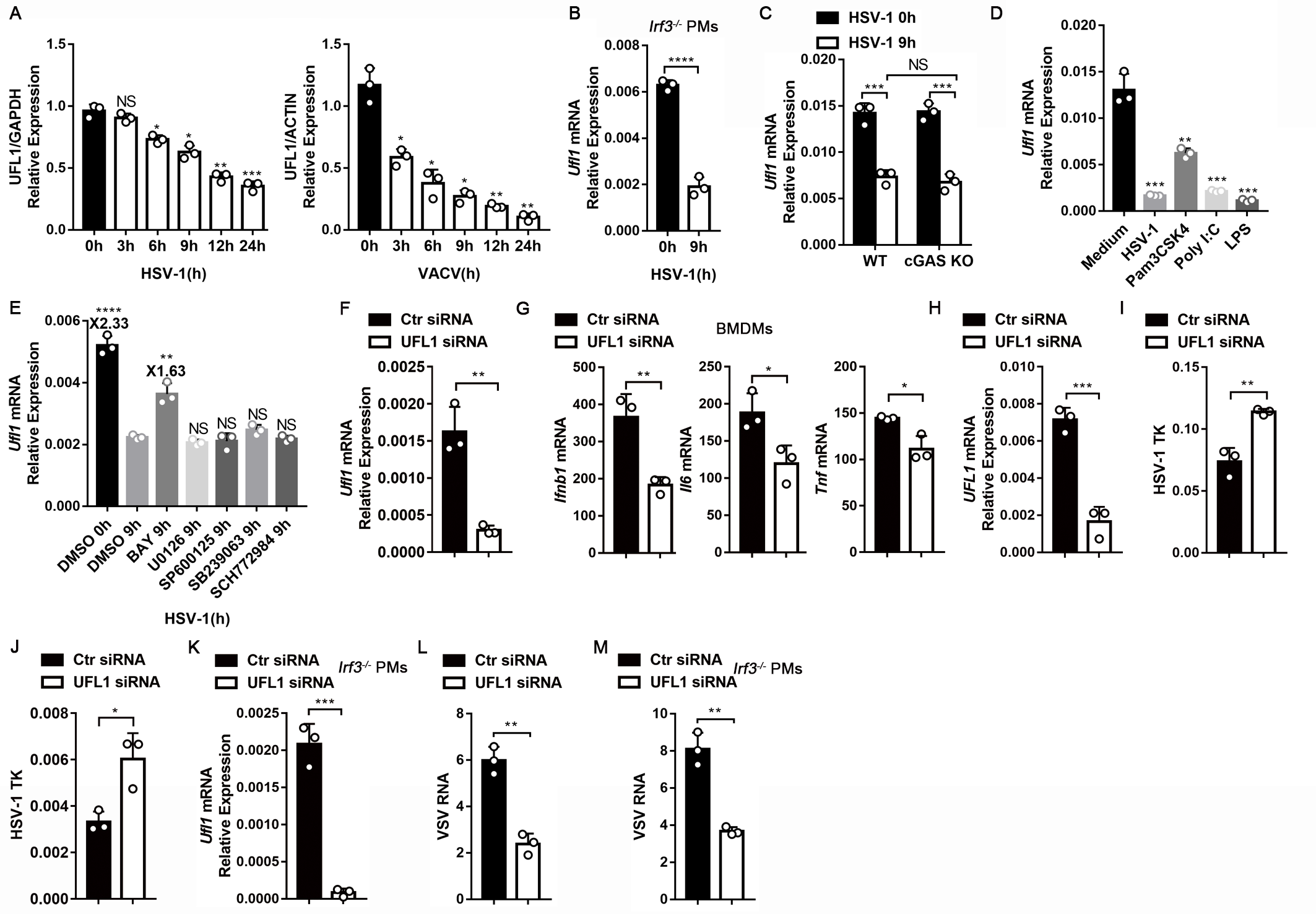

Supplement: Supplementary file 5 — Supplementary Figure 1 [file 41418_2022_1041_MOESM5_ESM.tif]

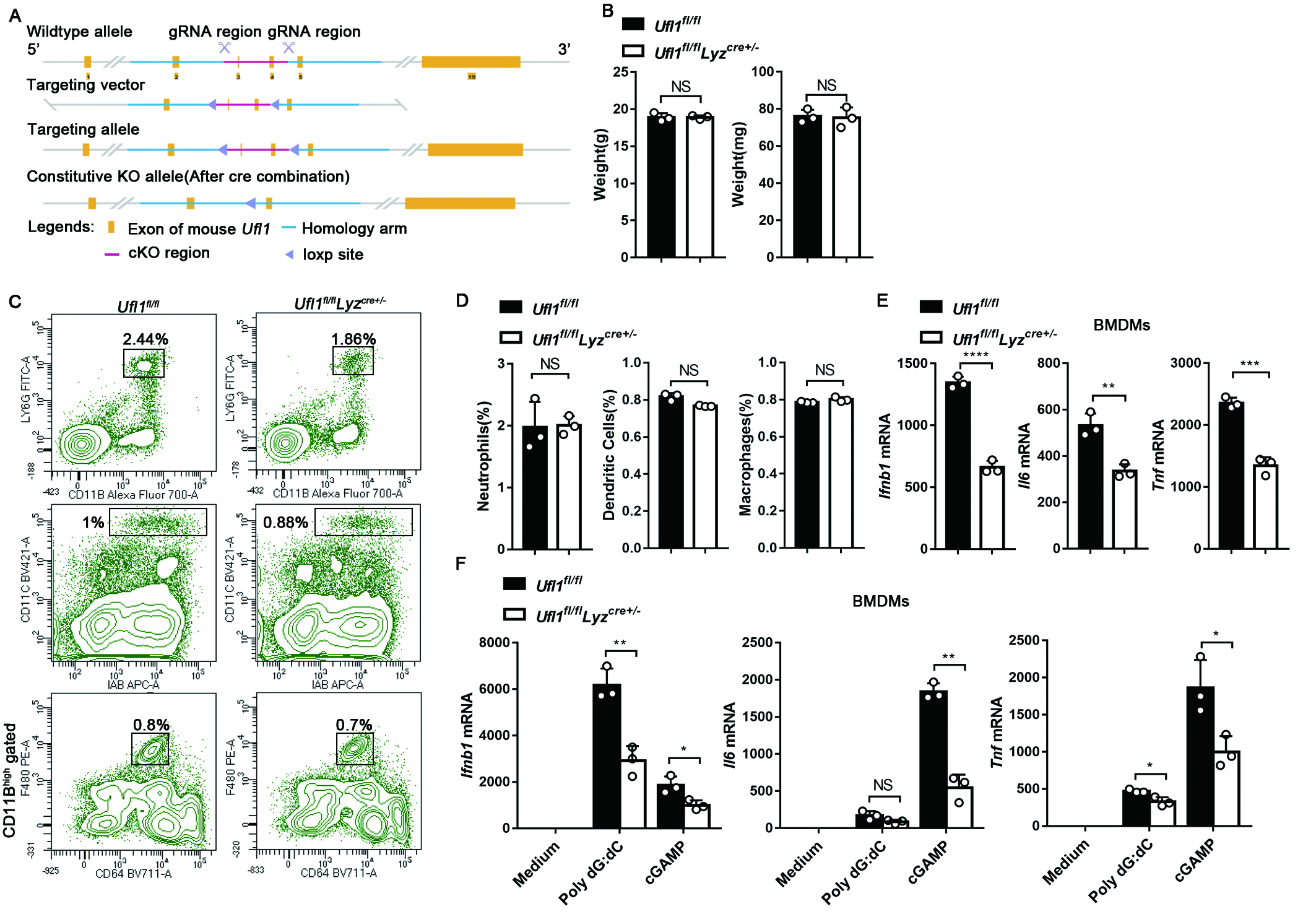

Supplement: Supplementary file 6 — Supplementary Figure 2 [file 41418_2022_1041_MOESM6_ESM.tif]

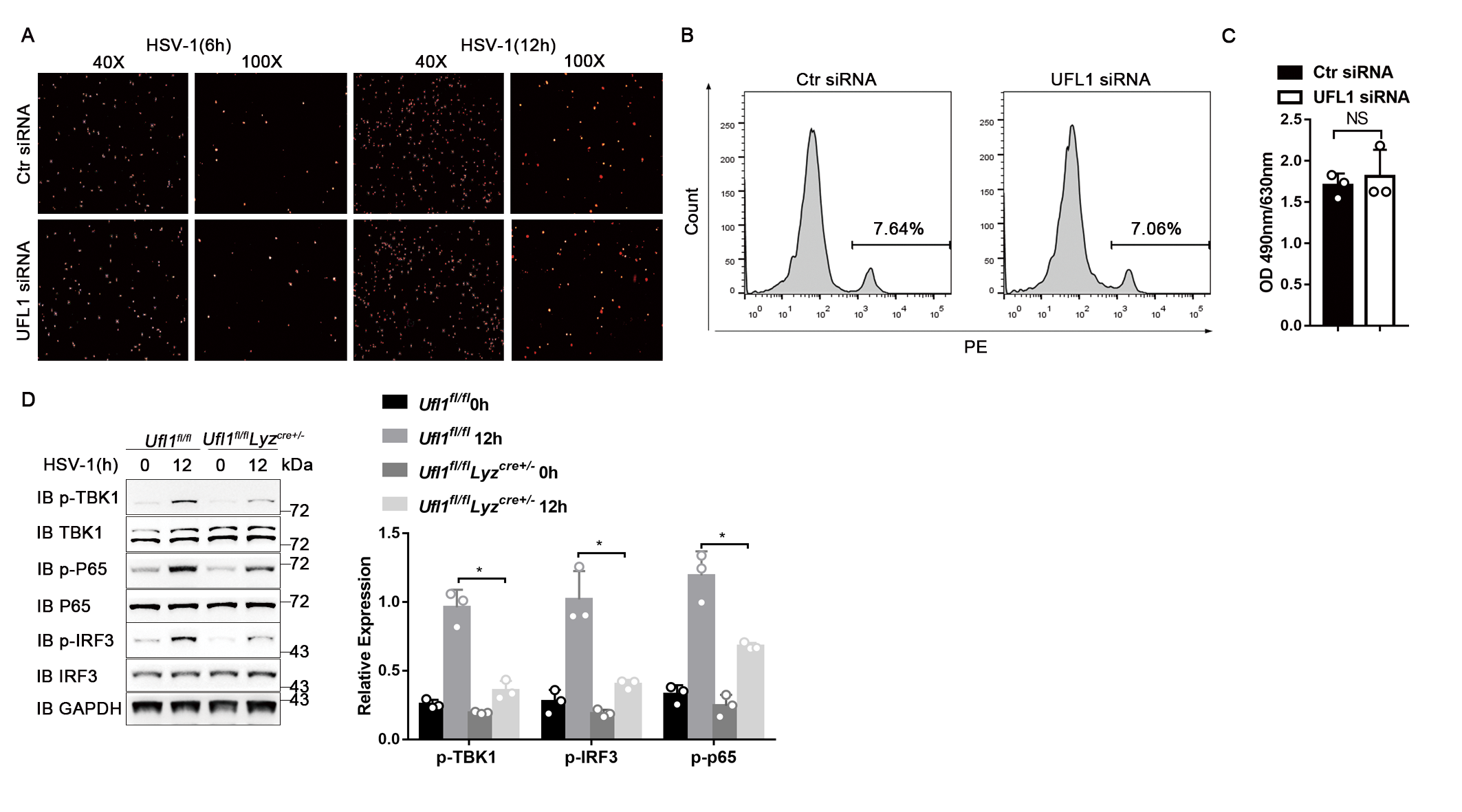

Supplement: Supplementary file 7 — Supplementary Figure 3 [file 41418_2022_1041_MOESM7_ESM.tif]

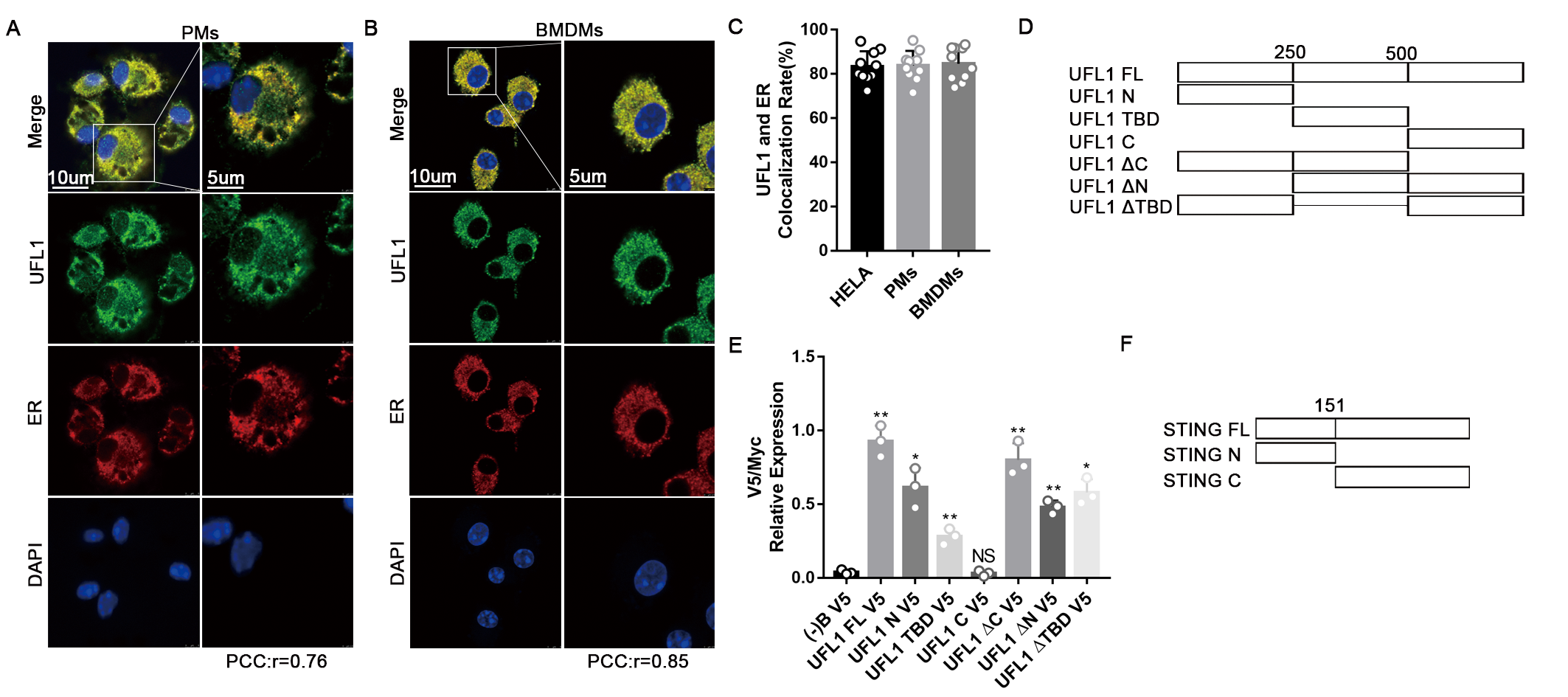

Supplement: Supplementary file 8 — Supplementary Figure 4 [file 41418_2022_1041_MOESM8_ESM.tif]

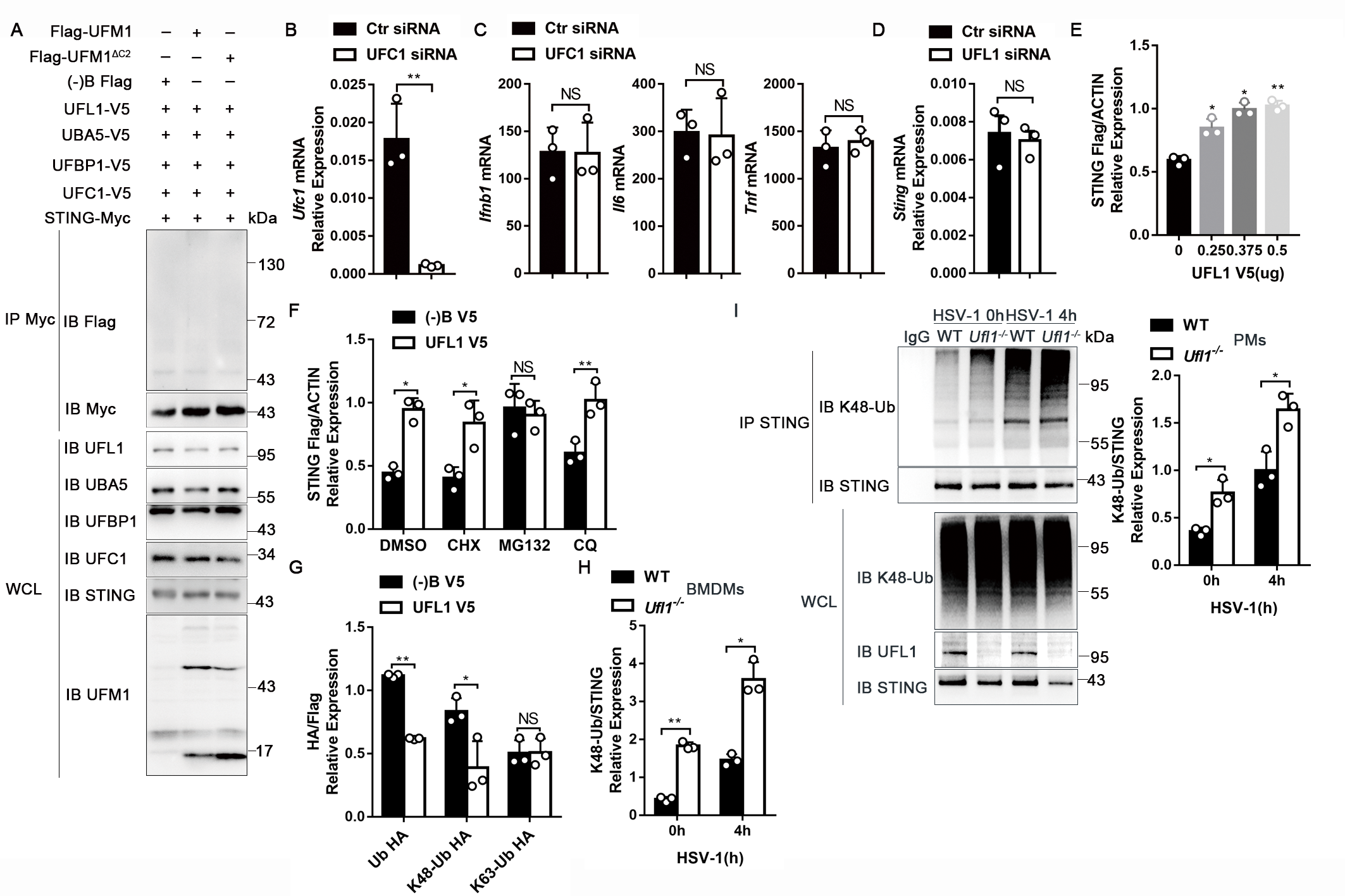

Supplement: Supplementary file 9 — Supplementary Figure 5 [file 41418_2022_1041_MOESM9_ESM.tif]

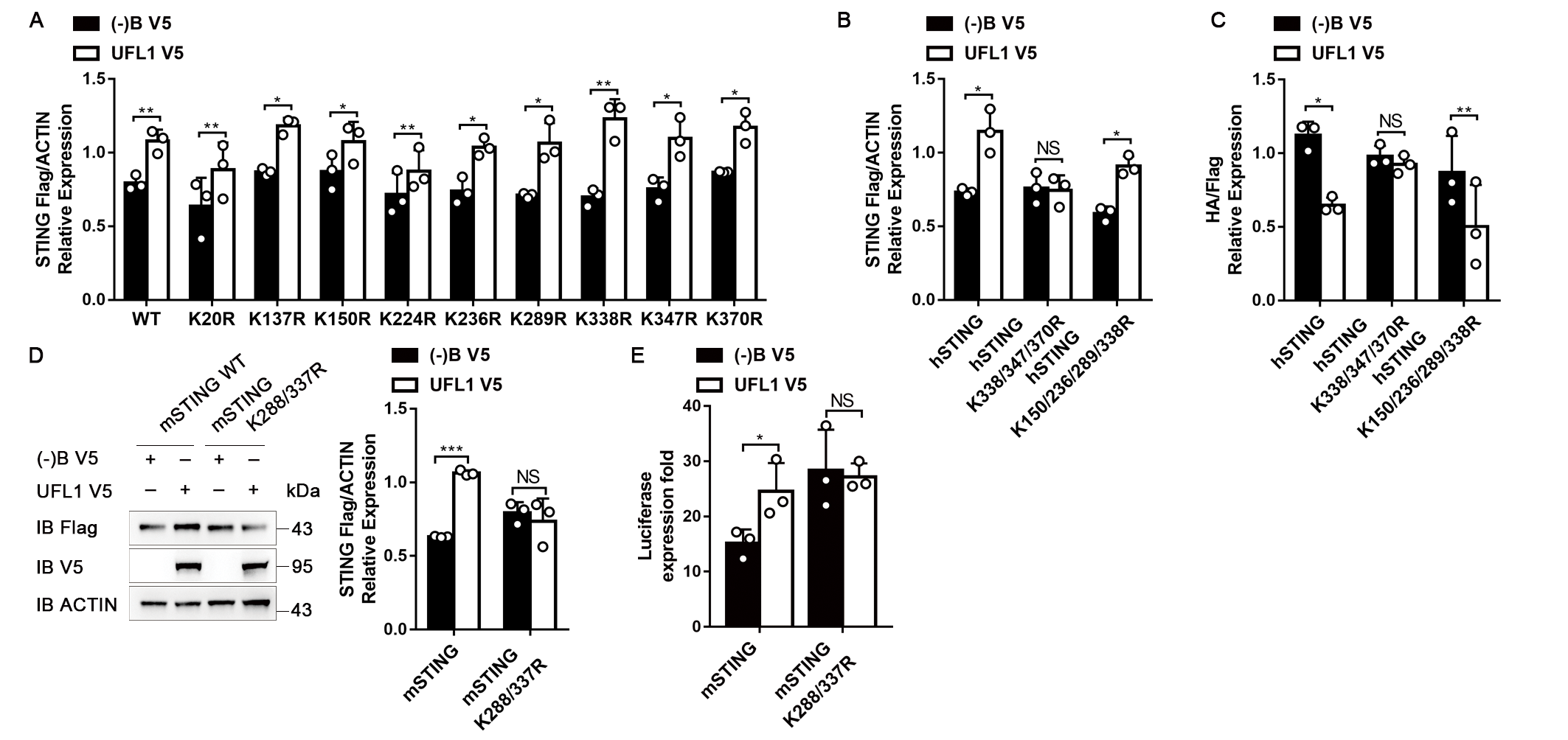

Supplement: Supplementary file 10 — Supplementary Figure 6 [file 41418_2022_1041_MOESM10_ESM.tif]

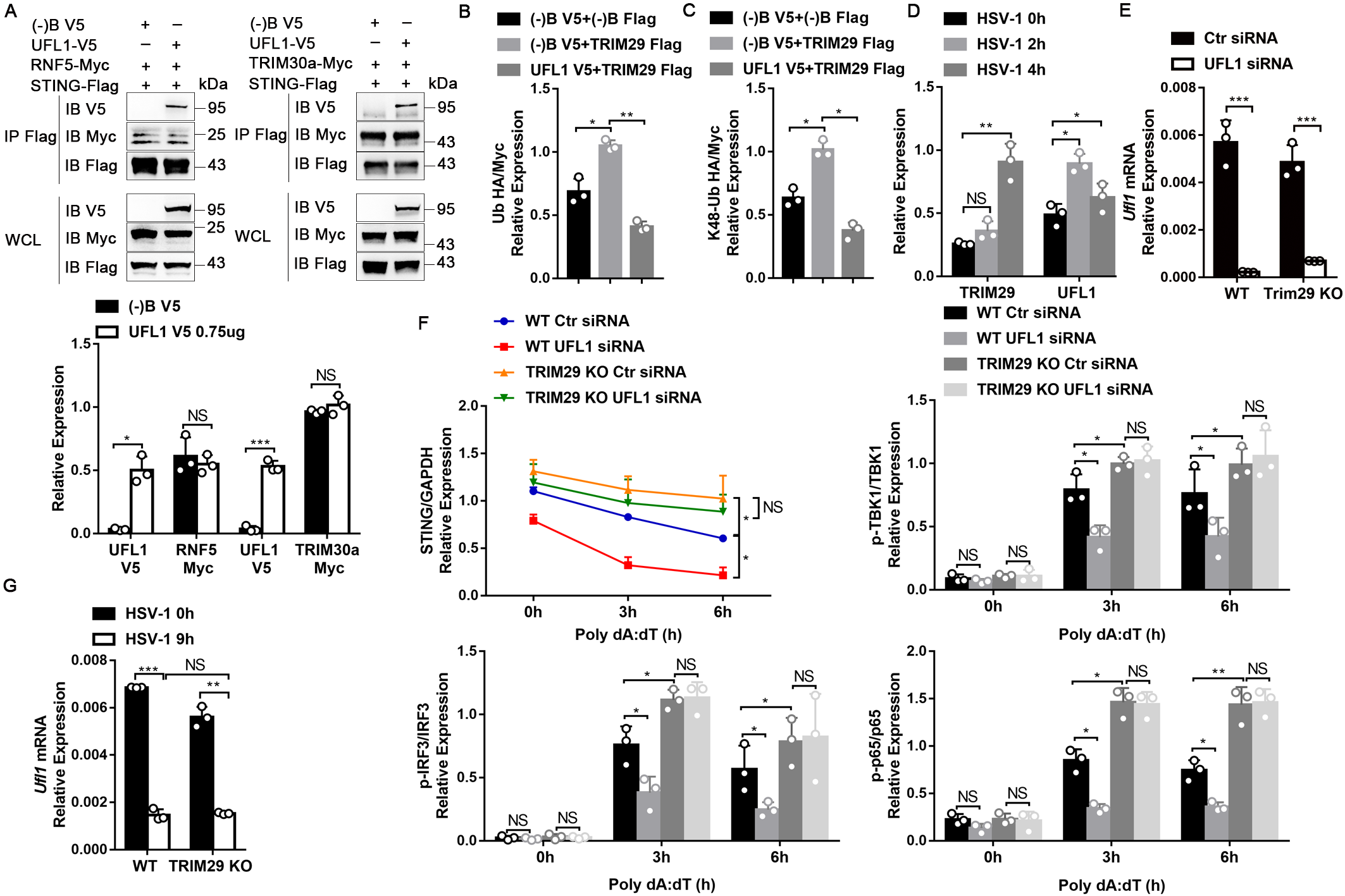

Supplement: Supplementary file 11 — Supplementary Figure 7 [file 41418_2022_1041_MOESM11_ESM.tif]
