# Supplementary material for: UFL1 promotes antiviral immune response by maintaining STING stability independent of UFMylation
Source: Cell Death Differ. 2022 Jul 23;30(1):16–26. doi: 10.1038/s41418-022-01041-9 (PMC9883236; doi:10.1038/s41418-022-01041-9)
Supplement: Supplementary file 12 — Supplemental Material-western blots [file 41418_2022_1041_MOESM12_ESM.docx]

**Supplemental Material**

**UFL1 Promotes Antiviral Immune Response by Maintaining STING Stability Independent of UFMylation**

**Figure 1B**

HSV-1 IB UFL1


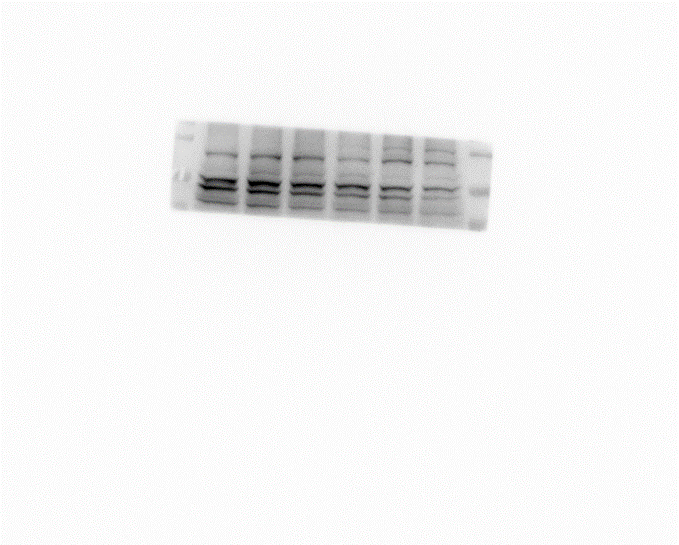


HSV-1 IB GAPDH


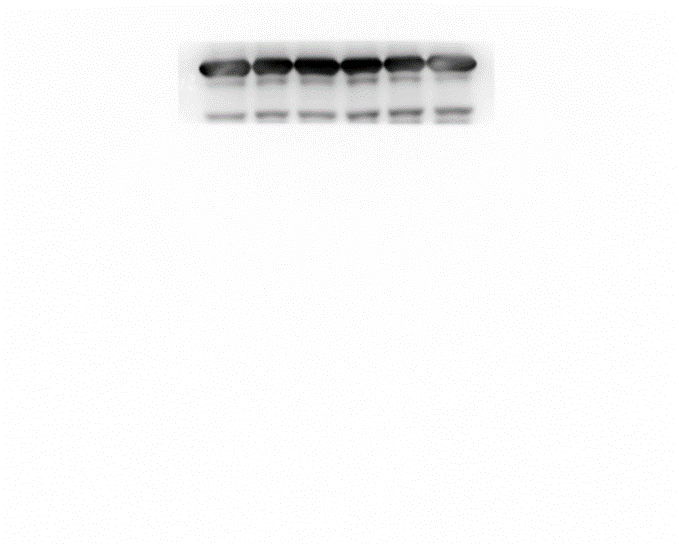


VACV IB UFL1





VACV IB β-actin





**Figure 3C**

IB UFL1





IB p-TBK1





IB TBK1





IB p-IKKβ





IB IKKβ





IB p-P65





IB P65





IB p-IRF3





IB IRF3





IB β-actin





**Figure 3D**

IB UFL1





IB p-TBK1





IB TBK1





IB p-IKKβ





IB IKKβ





IB p-P65





IB P65





IB p-IRF3





IB IRF3





IB β-actin





**Figure 4A**

IP V5 IB Flag


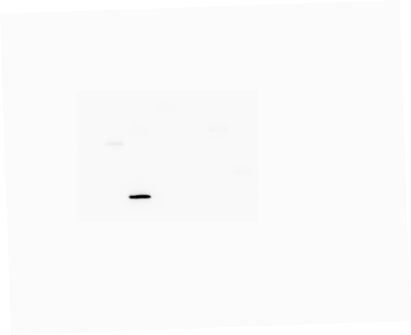


IP V5 IB V5


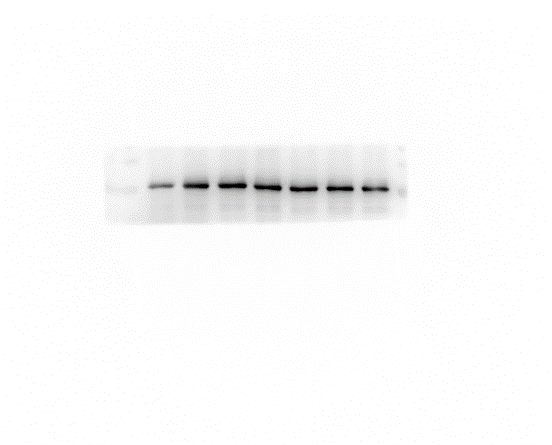


WCL IB Flag


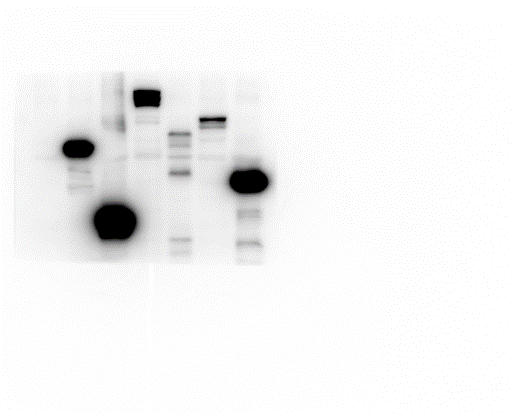


WCL IB V5


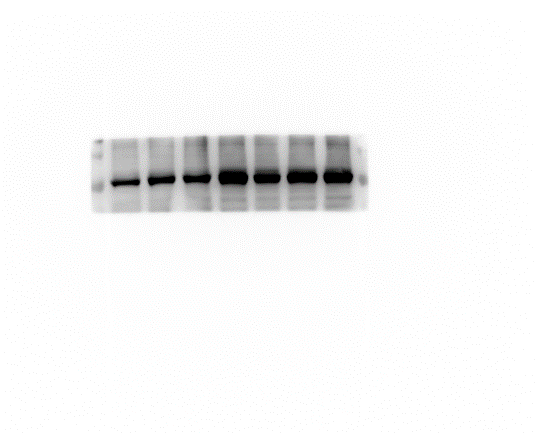


**Figure 4B**

UFL1+STING

IP Flag IB V5





IP Flag IB Flag





WCL IB V5





WCL IB Flag





UFL1+cGAS

IP Flag IB V5





IP Flag IB Flag





WCL IB V5


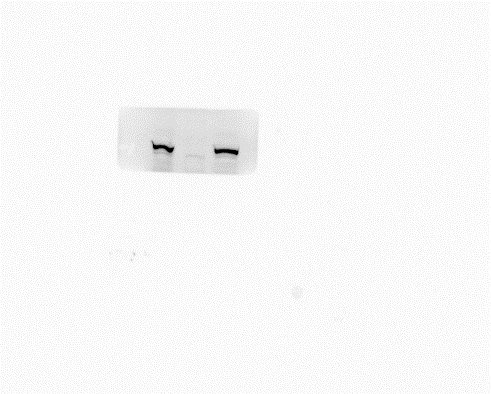


WCL IB Flag


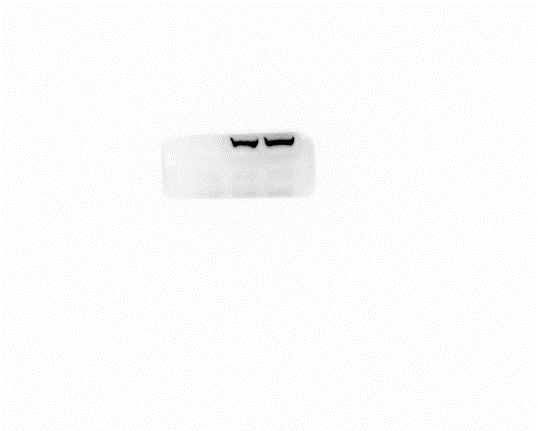


**Figure 4C**

IP STING IB UFL1





IP STING IB STING





WCL IB UFL1


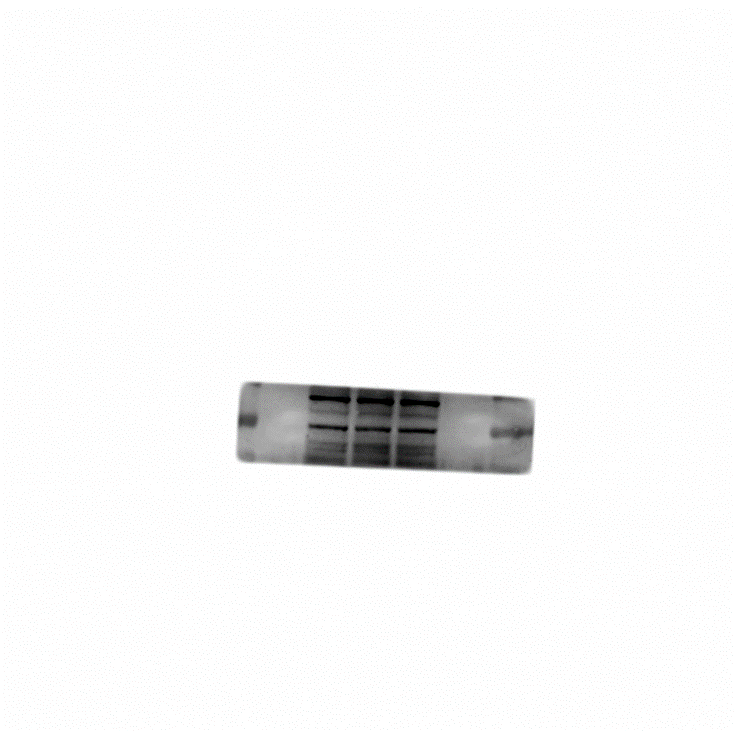


WCL IB STING





**Figure 4D**

IP STING IB UFL1





IP STING IB STING





WCL IB UFL1


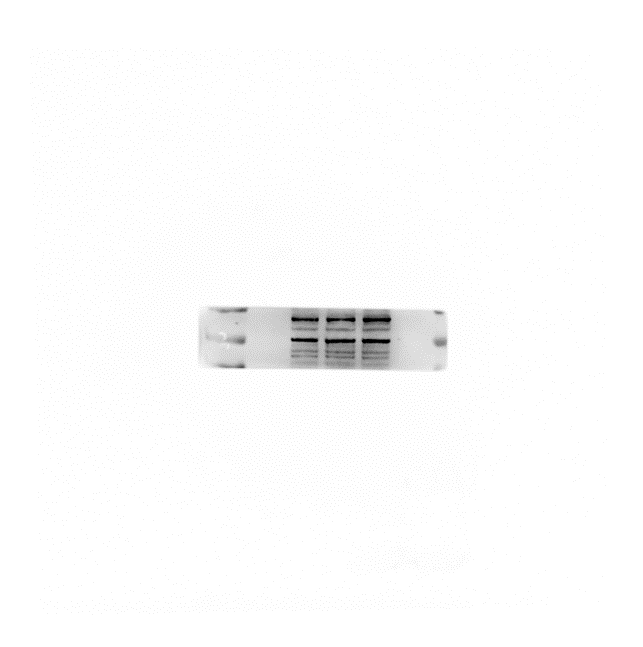


WCL IB STING


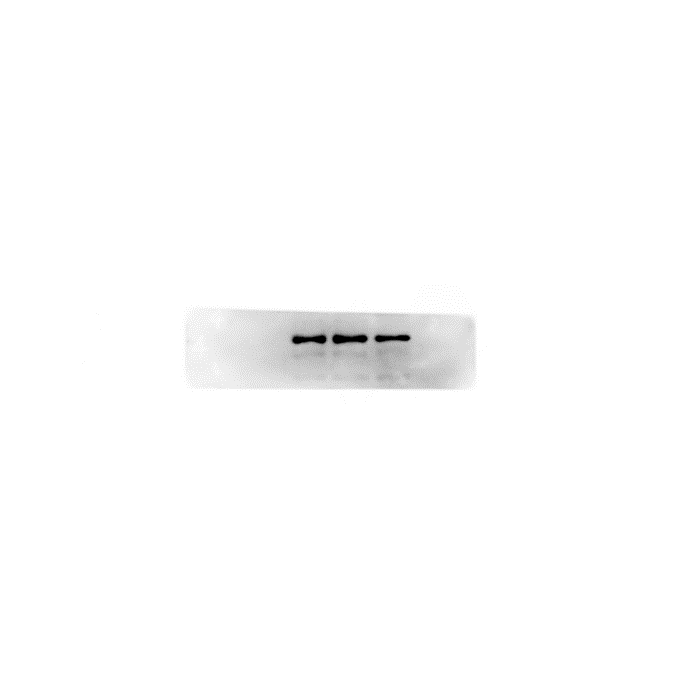


**Figure 4I**

IP Myc IB V5





IP Myc IB Myc





WCL IB V5





WCL IB Myc





**Figure 4J**

IP V5 IB Myc





IP V5 IB V5





WCL IB Myc





WCL IB V5





**Figure 5B**

IB STING


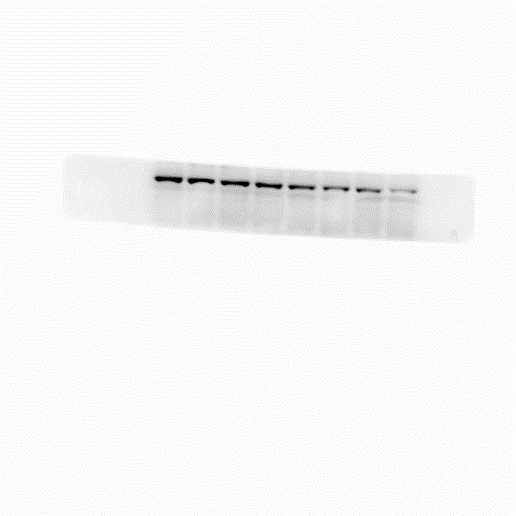


IB cGAS


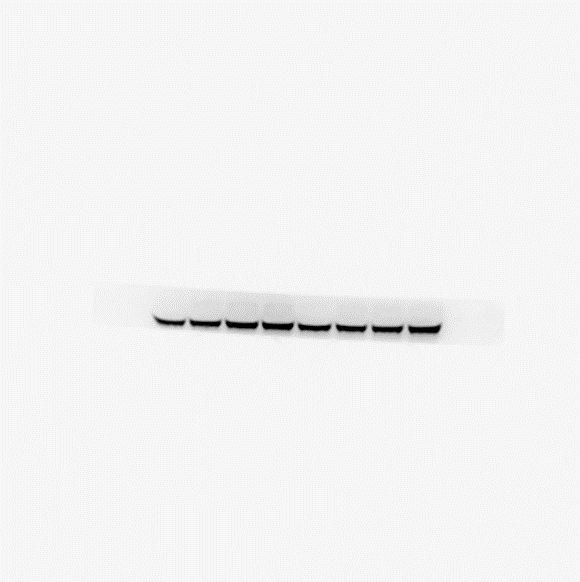


IB GAPDH





**Figure 5C**

IB STING

**
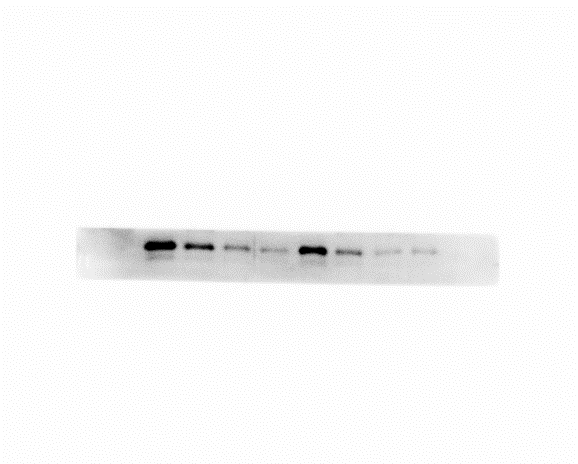
**

IB cGAS


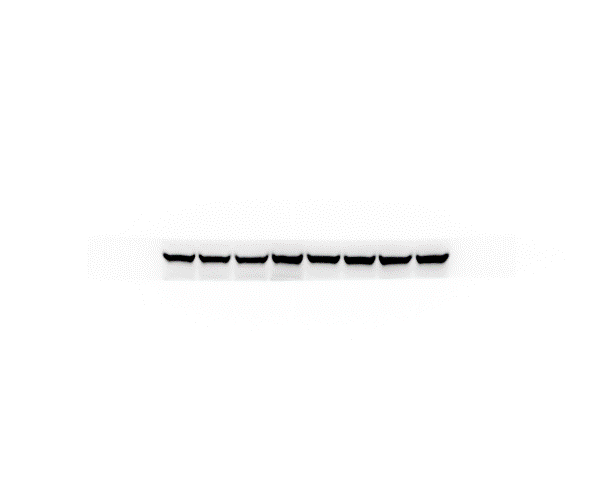


IB GAPDH





**Figure 5D**

IB V5


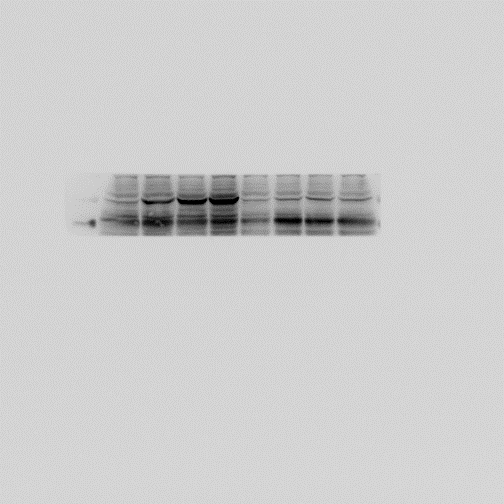


IB Flag


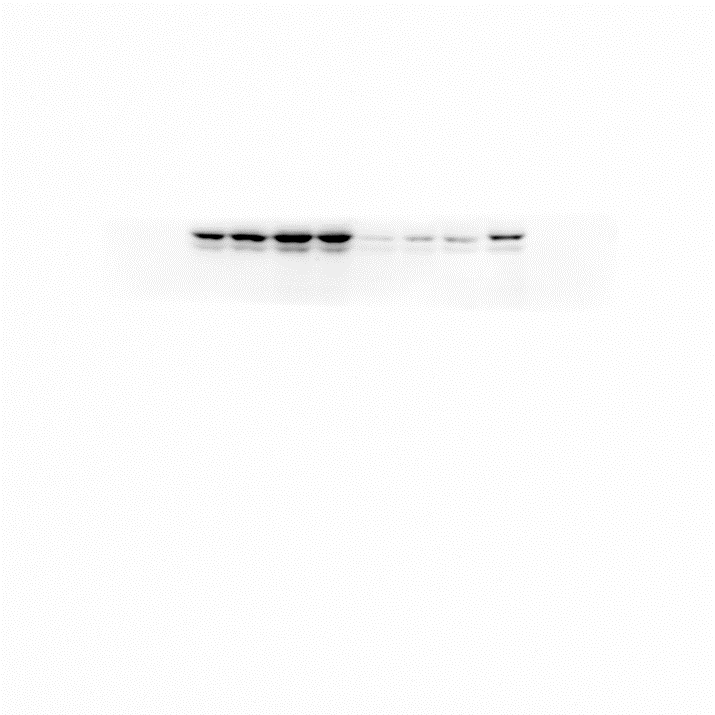


IB β-actin


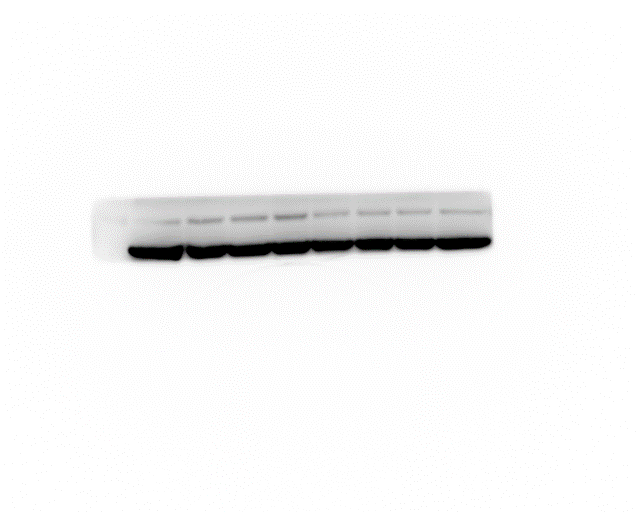


**Figure 5E**

IB Myc





IB V5


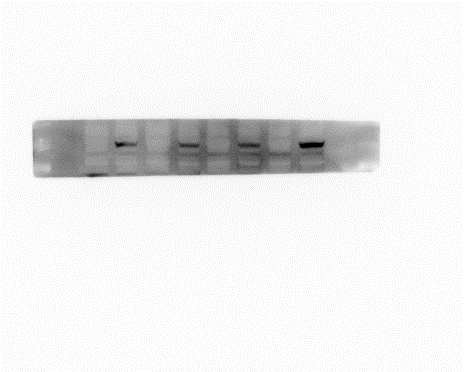


IB β-actin


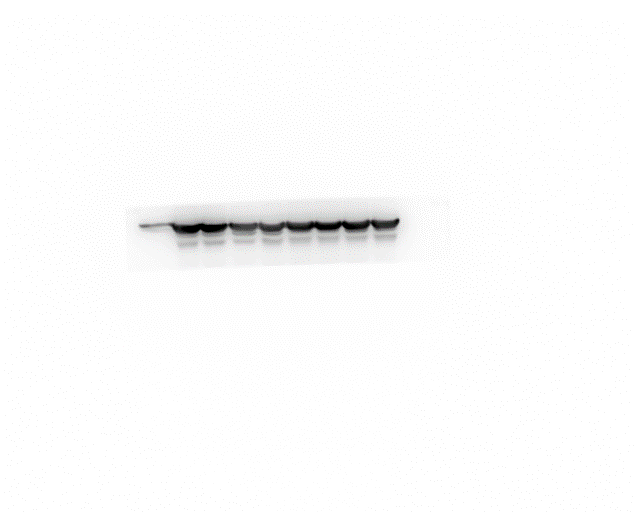


**Figure 5F**

DMSO IB STING





DMSO IB UFL1





DMSO IB β-actin





MG132 IB STING





MG132 IB UFL1





MG132 IB β-actin





CQ IB STING





CQ IB UFL1





CQ IB β-actin





**Figure 5G**

IP Myc IB HA





IP Myc IB Myc





WCL IB HA





WCL IB Myc





WCL IB V5





**Figure 5H**

IP STING IB K48-Ub





IP STING IB STING





WCL IB K48-Ub





WCL IB UFL1





WCL IB STING





**Figure 6A**

IB Flag







IB V5







IB β-actin


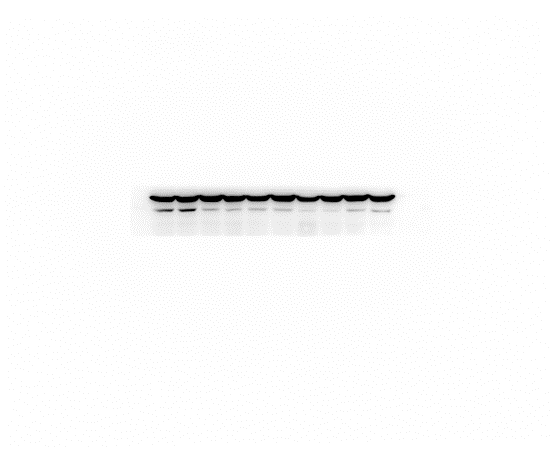

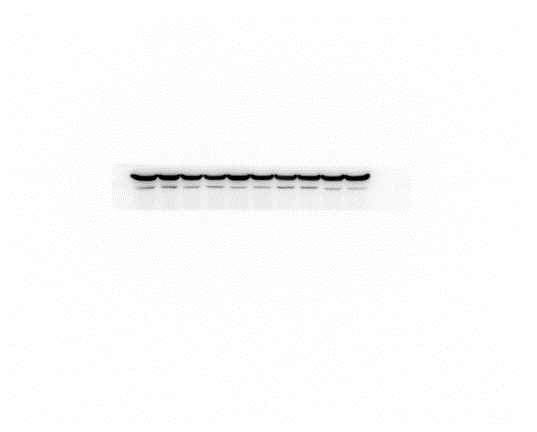


**Figure 6C**

IB Flag





IB V5





IB β-actin





**Figure 6E**

IP Flag IB HA





IP Flag IB Flag





WCL IB HA





WCL IB Flag





WCL IB V5





**Figure 7A**

IP Myc IB V5





IP Myc IB Flag





IP Myc IB Myc





WCL IB V5

WCL IB Flag

WCL IB Myc

**Figure 7C**

IP Myc IB HA

IP Myc IB Myc

WCL IB HA

WCL IB Myc

WCL IB V5

WCL IB Flag

**Figure 7D**

IP Myc IB HA

IP Myc IB Myc

WCL IB HA

WCL IB Myc

WCL IB V5

WCL IB Flag

**Figure 7E**

IP STING IB TRIM29

IP STING IB UFL1

IP STING IB STING

WCL IB TRIM29

WCL IB UFL1

WCL IB STING

**Figure 7G**

IB TRIM29

IB p-TBK1

IB TBK1

IB p-IRF3

IB IRF3

IB p-P65

IB P65

IB STING

IB GAPDH

**Supplementary Figure 3D**

IB P-TBK1

IB TBK1

IB P-IRF3

IB IRF3

IB P-P65

IB P65

IB GAPDH

**Supplementary Figure 5A**

IP Myc IB Flag

IP Myc IB Myc

WCL IB V5 (UFL1 UBA5 UFBP1)

WCL IB V5 (UFC1)

WCL IB Flag

WCL IB Myc

**Supplementary Figure 5E**

IP STING IB K48-Ub

IP STING IB STING

WCL IB K48-Ub

WCL IB UFL1

WCL IB STING

**Supplementary Figure 6A**

IB Flag

IB V5

IB β-actin

**Supplementary Figure 7A**

**STING+RNF5**

IP Flag IB V5

IP Flag IB Myc

IP Flag IB Flag

WCL IB V5

WCL IB Myc

WCL IB Flag

**STING+TRIM30a**

IP Flag IB V5

IP Flag IB Myc

IP Flag IB Flag

WCL IB V5

WCL IB Myc

WCL IB Flag

**Replicates of Immunoblot Data**

**Replicates of Fig.1**

**Figure 1B**

HSV-1 IB UFL1

HSV-1 IB GAPDH

VACV IB UFL1

VACV IB β-actin

**Replicates of Fig.3**

**Figure 3C**

IB p-TBK1

IB TBK1

IB p-IKKβ

IB IKKβ

IB p-P65

IB P65

IB p-IRF3

IB IRF3

IB β-actin

**Figure 3D**

IB p-TBK1

IB TBK1

IB p-IKKβ

IB IKKβ

IB p-P65

IB P65

IB p-IRF3

IB IRF3

IB β-actin

**Replicates of Fig.4**

**Figure 4A**

IP V5 IB Flag

IP V5 IB V5

WCL IB Flag

WCL IB V5

**Figure 4B**

UFL1+STING

IP Flag IB V5

IP Flag IB Flag

WCL IB V5

WCL IB Flag

UFL1+cGAS

IP Flag IB V5

IP Flag IB Flag

WCL IB V5

WCL IB Flag

**Figure 4C**

IP STING IB UFL1

IP STING IB STING

WCL IB UFL1

WCL IB STING

**Figure 4D**

IP STING IB UFL1

IP STING IB STING

WCL IB UFL1

WCL IB STING

**Figure 4I**

IP Myc IB V5

IP Myc IB Myc

WCL IB V5

WCL IB Myc

**Figure 4J**

IP V5 IB Myc

IP V5 IB V5

WCL IB Myc

WCL IB V5

**Replicates of Fig.5**

**Figure 5B**

IB STING

IB cGAS

IB GAPDH

**Figure 5C**

IB STING

IB cGAS

IB GAPDH

**Figure 5D**

IB V5

IB Flag

IB β-actin

**Figure 5E**

IB Myc

IB V5

IB β-actin

**Figure 5F**

DMSO IB STING

DMSO IB UFL1

DMSO IB β-actin

MG132 IB STING

MG132 IB UFL1

MG132 IB β-actin

CQ IB STING

CQ IB UFL1

CQ IB β-actin

**Figure 5G**

IP Myc IB HA

IP Myc IB Myc

WCL IB HA

WCL IB Myc

WCL IB V5

**Figure 5H**

IP STING IB K48-Ub

IP STING IB STING

WCL IB K48-Ub

WCL IB UFL1

WCL IB STING

**Replicates of Fig.6**

**Figure 6A**

IB Flag 1

IB V5 1

IB β-actin 1

IB Flag 2

IB V5 2

IB β-actin 2

**Figure 6C**

IB Flag

IB V5

IB β-actin

**Figure 6E**

IP Flag IB HA

IP Flag IB Flag

WCL IB HA

WCL IB Flag

WCL IB V5

**Replicates of Fig.7**

**Figure 7A**

IP Myc IB V5

IP Myc IB Flag

IP Myc IB Myc

WCL IB V5

WCL IB Flag

WCL IB Myc

**Figure 7C**

IP Myc IB HA

IP Myc IB Myc

WCL IB HA

WCL IB Myc

WCL IB V5

WCL IB Flag

**Figure 7D**

IP Myc IB HA

IP Myc IB Myc

WCL IB HA

WCL IB Myc

WCL IB V5

WCL IB Flag

**Figure 7E**

IP STING IB TRIM29

IP STING IB UFL1

IP STING IB STING

WCL IB TRIM29

WCL IB UFL1

WCL IB STING

**Figure 7G**

IB TRIM29

IB p-TBK1

IB TBK1

IB p-IRF3

IB IRF3

IB p-P65

IB P65

IB STING

IB GAPDH

**Replicates of Supplementary Figure 3D**

**Supplementary Figure 3D**

IB p-TBK1

IB TBK1

IB p-IRF3

IB IRF3

IB p-P65

IB P65

IB GAPDH

**Replicates of Supplementary Figure 5**

**Supplementary Figure 5A**

IP Myc IB Flag

IP Myc IB Myc

WCL IB V5 (UBA5)

WCL IB V5 (UFL1)

WCL IB V5 (UFC1)

WCL IB V5 (UFBP1)

WCL IB Flag

WCL IB Myc

**Supplementary Figure 5E**

IP STING IB K48-Ub

IP STING IB STING

WCL IB K48-Ub

WCL IB UFL1

WCL IB STING

**Replicates of Supplementary Figure 6**

**Supplementary Figure 6A**

IB Flag

IB V5

IB β-actin

**Replicates of Supplementary Figure 7**

**Supplementary Figure 7A**

**STING+RNF5**

IP Flag IB V5

IP Flag IB Myc

IP Flag IB Flag

WCL IB V5

WCL IB Myc

WCL IB Flag

**STING+TRIM30a**

IP Flag IB V5

IP Flag IB Myc

IP Flag IB Flag

WCL IB V5

WCL IB Myc

WCL IB Flag
